# APOE4 and sedentary lifestyle synergistically impair neurovascular function in the visual cortex of awake mice

**DOI:** 10.1038/s42003-025-07585-z

**Published:** 2025-01-29

**Authors:** Silvia Anderle, Orla Bonnar, Joseph Henderson, Kira Shaw, Andre M. Chagas, Letitia McMullan, Alexandra Webber, Kirsty McGowan, Sarah L. King, Catherine N. Hall

**Affiliations:** 1 School of Psychology and Sussex Neuroscience, University of Sussex, Brighton, UK; 2 Department of Neuroscience, Physiology and Pharmacology, University College London, London, UK; 3 MassGeneral Institute for Neurodegenerative Disease, Massachusetts General Hospital and Harvard Medical School, Boston, USA; 4 Department of Clinical Neurosciences, University of Cambridge, Cambridge, UK

**Keywords:** Neuro-vascular interactions, Alzheimer's disease

## Abstract

Reduced cerebral blood flow occurs early in the development of Alzheimer’s disease (AD), but the factors producing this reduction are unknown. Here, we ask whether genetic and lifestyle risk factors for AD—the ε4 allele of the Apolipoprotein (APOE) gene, and physical activity—can together produce this reduction in cerebral blood flow which leads eventually to AD. Using in vivo two-photon microscopy and haemodynamic measures, we record neurovascular function from the visual cortex of physically active or sedentary mice expressing APOE3 and APOE4 in place of murine APOE. Energy supply and demand are mismatched in APOE4 mice, with smaller increases in cerebral blood flow, blood volume and blood oxygenation occurring during neuronal activation as blood vessels frequently fail to dilate. Exercise dose-dependently overall improves neurovascular function, with an increased impact of exercise apparent after longer exposure times. Several haemodynamic measures show a larger beneficial effect of exercise in APOE4 vs. APOE3 mice. Thus, APOE4 genotype in conjunction with sedentary behaviour produces the worst neurovascular function. Promotion of physical activity may therefore be particularly important to improve cerebrovascular function and reduce dementia risk in APOE4 carriers.

## Abstract (web summary)

Lifestyle matters for genetic Alzheimer’s disease risk: In vivo imaging of mouse blood vessels and neurons shows that APOE4 reduces neurovascular function most in an already-stressed system, when neurons are active and mice do less exercise.

## Introduction

Changes in cerebral blood flow are one of the earliest signs of Alzheimer’s disease (AD), appearing many years prior to the onset of cognitive symptoms^1^, suggesting a link between vascular dysfunction and AD development. This link is particularly evident in patients carrying the most common genetic risk factor for late onset Alzheimer’s disease: apolipoprotein E-ε4 (APOE4), who show a rapid decline in cerebral blood flow in old age, before developing AD^2^. Heterozygote APOE4 carriers have a four times higher risk of developing AD, rising to a fifteen times higher risk in those who possess two copies of the APOE4 gene^3,4^ and recent work suggests that by 80 years almost all APOE4 homozygotes will have developed AD pathology and symptoms^5^. Understanding how APOE4 imposes increased risk of AD is important for developing therapeutic interventions that could reduce this risk.

APOE4 is known to affect the brain vasculature, disrupting blood brain barrier (BBB) function^6^, likely via LRP-1 and the cyclophilin A-matrix metalloproteinase 9 (CypA/MMP9) pathway^7,8^. However, it is much less clear how APOE genotype contributes to the decrease in cerebral blood flow that is observed before the onset of dementia. APOE4 could reduce blood flow via a reduction in vascular density, as mouse and human studies found APOE4 carriers to have reduced vascular density in the brain^9,10^ and in the retina^11^. Alternatively, a reduction in cerebral blood flow could also be produced by a failure to match energy supply and demand via dysfunctional neurovascular coupling—i.e. a failure of increases in neuronal activity to increase local blood vessel diameter and blood flow.

However, previous studies have found mixed results as to the impact of APOE4 on neurovascular coupling. Studies in acutely-imaged, anaesthetised mice found dramatically reduced blood flow responses in APOE4-TR mice^9,10^. Conversely, we found only subtle alterations in neurovascular function in awake mice which had fully recovered from cranial window surgery and were provided with an exercise wheel in their home cage^12^. A likely reason for this discrepancy is the increased sensitivity of APOE4 carriers to pathological insults such as anaesthesia^13,14^, inflammation^15^, and/or lifestyle differences^12^.

Increased activity and exercise are well established to modulate brain function, increasing cerebral blood flow^16–19^, augmenting vascular density^20,21^ and promoting neurogenesis and plasticity^22^. APOE4 carriers might particularly benefit from exercise, as APOE4 carriers who performed physical exercise showed better maintenance of cognitive function^23,24^ and increased hippocampal blood flow^25^ compared to non-carriers subject to exercise.

Together, the increased pathological impact of APOE4 genotype during acute surgery, and the potential sensitivity of APOE4 carriers to exercise, suggests that APOE4 is not a necessary driver of severe neurovascular dysfunction, but needs to interact with other negative stressors, such as lack of exercise, to impact cerebral blood flow and the brain’s energy supply and demand balance. To interrogate this possibility, we tested whether the effect of APOE4 genotype on neurovascular function is modulated by the activity level of the mice, such that neurovascular deficits are worst in sedentary APOE4-carrying mice, with function improved by increased physical activity. We measured various aspects of neuronal and vascular function in the visual cortex of mice expressing either the human APOE3 or APOE4 gene in place of murine APOE, after mice had been housed with or without an exercise wheel for 1-2 (imaging timepoint 1) or 4-5 (imaging timepoint 2) months. Provision of an exercise wheel increased mouse activity levels, and improved several aspects of cerebrovascular function, including capillary density, regional blood oxygen levels, arteriolar vasomotion, and neuronal activity-induced vessel dilations. Furthermore, APOE4 genotype frequently interacted with the effect of exercise, with APOE4 sedentary mice showing the weakest neuronal and vascular function.

Thus, increased physical activity protected against the poor neurovascular function of sedentary APOE4-TR mice. These results provide a potential explanation for previous divergent results in the literature, and suggest that promoting physical exercise may be important to increase resilience of human APOE4 carriers to dementia by improving vascular function.

## Results

### Experimental timeline and imaging set up

**Fig. 1:**
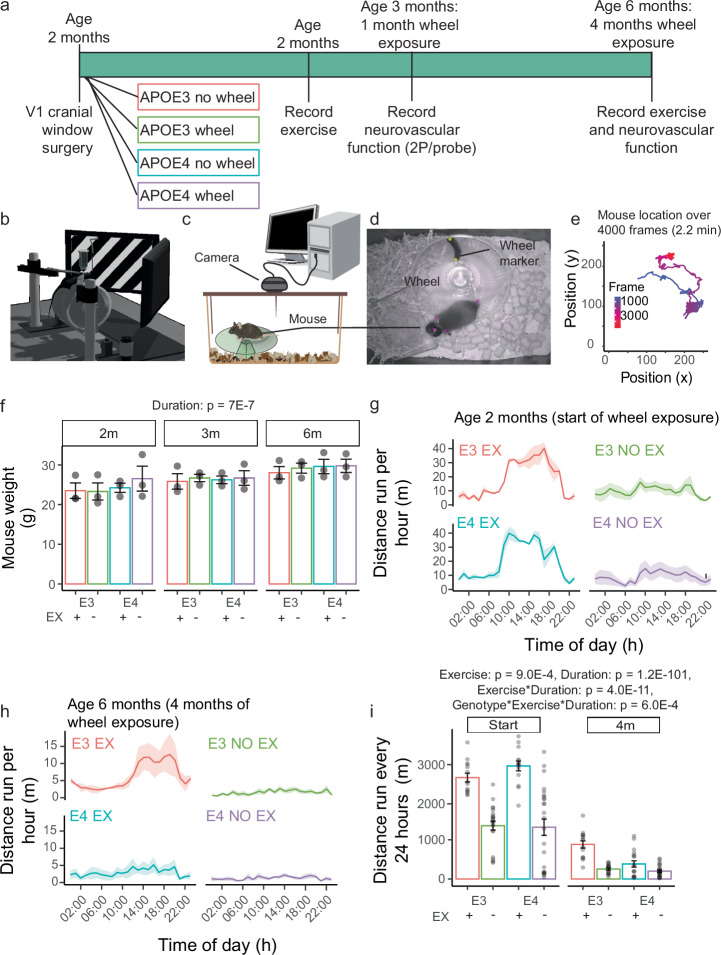
Mice provided with an exercise wheel are more active. **a** Experimental timeline, showing experimental groups (APOE genotype, and wheel access), time of cranial window implantation and data collection. **b** Schematic of the in vivo recording set up (D. Grijseels, CC-BY^12^). The mouse was head fixed to two metal arms and was freely walking on a rotating cylinder containing a rotary encoder while facing two screens that presented a visual stimulation (drifting grating). Recordings of net haemodynamic measures were obtained with an Oxy-CBF probe and measurements of neurovascular function were recorded with two-photon microscopy. **c** Activity levels were recorded from the home cage of the mouse using a USB infrared camera mounted in a 3D printed support positioned on the top of the cage. The camera was connected to a PC where videos are recorded using Bonsai.rx. The schematic was created using images of a monitor^83^, mouse^84^ and cage^85^, available with Creative Commons licences. **d** Videos were analysed using DeepLabCut. A deep learning network was trained to recognize mouse body parts (coloured dots on cranial window, right head plate, left head plate and tail) and a dark marker placed on the wheel (wheel marker- yellow dots indicate inner mark and outer mark). **e** Mouse position in each frame from one representative recording, tracked using DeepLabCut, allowing calculation of the distance travelled by the mouse (from mouse displacement plus movement of wheel marker). **f** Mouse weights increased across the experiment but were not affected by genotype or exercise group. *N* = 3 mice per group. Mean distance run per hour for each experimental group at (**g**) the onset of wheel exposure and (**h**) after 4 months of wheel access. **i** Distance run every 24 h for each mouse at the start and after 4 months of wheel access. Dots are daily distances travelled by each mouse during each day of the recording session. Bars and error bars: mean +/- SEM. Daily distances: *N* = 14–26 from *N* = 3–6 mice. For details on *N* and statistical outputs see Supplementary Table 1 in Supplementary Data.

In order to assess how APOE genotype affected neurovascular function, we crossed mice homozygous for targeted replacement of murine APOE with human APOE3 or APOE4 with mice that expressed the genetically encoded calcium indicator GCaMP6f in excitatory neurons. 2-month-old mice were implanted with a cranial window over the visual cortex (V1) and then housed with or without an exercise wheel in their home cage. Activity levels were recorded in the home cage when the mice were 2 and 6 months old (Fig. 1a, c–e), and neurovascular function was measured at 3 months and 6 months of age, after 1 and 4 months of differential wheel exposure, respectively (Fig. 1a), while head-fixed on a rotating cylinder, which measured their voluntary locomotion with a rotary encoder. Computer monitors presented the mouse with visual stimuli (drifting gratings; Fig. 1b) so that neurovascular activity in the dark or in response to visual stimuli could be measured using two-photon (2 P) microscopy and a combined haemoglobin spectrometer and laser doppler flowmeter (Oxy-CBF; Fig. 1b). Mouse weights increased over the experiment (Fig. 1f) but were not different across the genotypes or exercise groups.

### Provision of a wheel increases exercise

Mouse activity levels were recorded for 72–120 h continually, using an infrared camera over the home cage (Bonsai.rx; Fig. 1c). The mouse’s location was tracked every frame (30 frames/second) using DeepLabCut (Fig. 1d, e) and from this, the average distance travelled each hour of the day (Fig. 1g, h) and the total distance travelled each day (Fig. 1i) were calculated. Mice were more active at 2 months of age, at the start of the experiment, compared with 6 months of age, after 4 months of wheel access (Fig. 1g–i), in line with previous reports^26,27^. Provision of an exercise wheel successfully increased activity levels, though the increase was smaller after 4 months of wheel access than at the start of the experiment, especially in APOE4 mice (Fig. 1g–i, Supplementary Fig. 1).

### Exercise increases capillary density and increases baseline sO2 in older mice

**Fig. 2:**
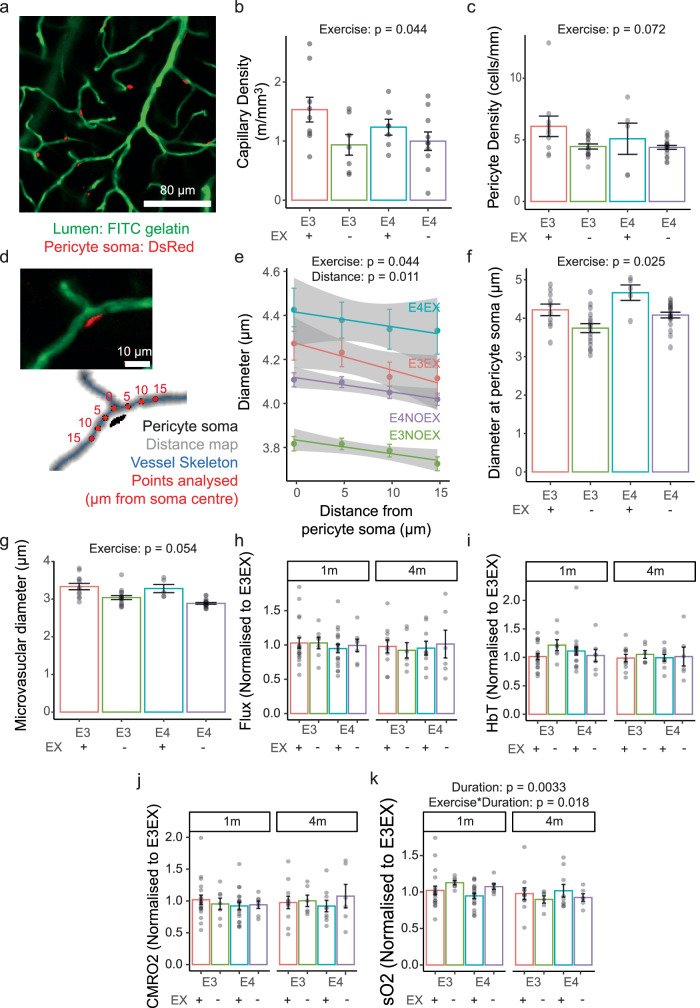
Sedentary mice have lower capillary density, lower pericyte density, more constricted capillaries and a reduction in baseline saturated oxygen levels over time. **a** Example of z-projected images of FITC-gelatine perfused vessels (green) and NG2-DsRed pericytes (red) in the visual cortex (image from E3EX mouse). Scale bar: 80 µm. Projected depth: 100 µm. **b** Exercise increased capillary density in V1 (for vessels <7 µm in diameter). Dots are average density per brain slice, statistical analysis was conducted on mean values per slice (*N* = 7–10 sections from *N* = 3–4 mice). **c** Pericyte density trends towards being higher in active mice. Statistical analysis was conducted on mean values per slice (*N* = 5–19 sections from *N* = 2–4 mice). **d** Example image of capillary showing pericyte (left, top: green: FITC gelatine, red: DsRed positive pericyte soma); and processed image showing skeleton (blue) and distance map coding distance of each pixel from the parenchyma (centre of vessel is darkest grey). Location of diameter measurements at varying distances from the pericyte soma are marked with red dots. **e** Capillary diameter as a function of distance from pericyte soma (soma is at 0 μm). **f** Capillary diameter at the pericyte soma is reduced in sedentary mice (*N* = 154–1189 pericytes from *N* = 2–4 mice). **g** Capillary diameter across the whole vascular network. Sedentary mice trend towards a smaller average diameter than active mice. (*N* = 5–19 images from *N* = 2–5 mice). Genotype, exercise and experiment duration did not affect net flux (**h**), HbT (**i**) or CMRO2 (**j**) at baseline. **k** sO2 was significantly reduced with age but was higher in mice that exercised. Dots show average per animal (*N* mice: 6–19). Bars and error bars: mean +/- SEM. For details of *N* and statistical outputs see Supplementary Table 2 in Supplementary Data.

We previously found that APOE4 genotype did not affect capillary density in mice with access to an exercise wheel^12^, though other studies found a significant reduction in capillary density in APOE4 mice^9,10^ and in the retina of human APOE4 carriers^11^. Because exercise can also modify capillary density^21^, we measured capillary density in post-mortem tissue from the visual cortex of a separate cohort of 3-month-old NG2-DsRed x APOE-TR mice perfused with a fluorescent gelatine to label the vasculature (Fig. 2a). One group of mice had 1–1.5 months of wheel access, while the other had no wheel access. Some of the data from the mice with wheel access was previously published^12^, as detailed in Methods. Exercise increased capillary density (Fig. 2b), but there was no effect of genotype on capillary density nor an interaction between genotype and exercise on capillary density. Thus, APOE4 genotype does not affect capillary density in visual cortex in active or sedentary mice. We also measured pericyte density by counting the number of DsRed positive soma on vessels. Pericyte number was not significantly affected by exercise level, though there was a trend to a lower pericyte density in sedentary mice (Fig. 2c). We also analysed capillary diameters across the microvascular network and as a function of distance from pericyte soma (Fig. 2d). Pericytes usually provide dilatory tone to capillaries, especially near the pericyte soma^28^, but capillaries of sedentary mice were significantly narrower near pericyte cell bodies, compared to more physically active mice (Fig. 2e, f). Across the whole vascular network, there was a trend towards narrower capillaries in sedentary vs. physically active mice (Fig. 2g). Overall, these results suggest that exercise may be beneficial in increasing capillary diameter by relaxing pericytes, and by increasing microvascular and, possibly, pericyte density.

However, anatomical measures such as vascular density, do not inform whether those vessels are functioning correctly. To probe neurovascular function, in vivo haemodynamic measures (cerebral blood flux, speed; blood oxygen saturation (sO2), total haemoglobin (HbT) and the cerebral rate of metabolic oxygen consumption (CMRO2)) were recorded using a combined haemoglobin spectrometer and laser doppler flowmeter in visual cortex. Although we found that levels of locomotion during the Oxy-CBF recordings were similar between groups (Supplementary Fig. 2a), baseline data was averaged across periods of rest to exclude any confounding effect of locomotion-induced changes in haemodynamics.

In the absence of visual stimulation, baseline cerebral blood flux (Fig. 2h), speed (Supplementary Fig. 2b), HbT, which reflects blood volume (Fig. 2i), and CMRO2 (Fig. 2j) showed no effect of APOE genotype or exercise. On the other hand, sO2 values were significantly decreased in sedentary mice at 6 months of age (4 months since the differential exercise exposure) but were unaffected by APOE genotype (Fig. 2k). These data suggest that lower capillary densities and smaller capillary diameters due to sedentary lifestyle may lower sO2 values over time. Measuring haemodynamic function and capillary density in the same mice would directly test this conclusion, but this was not possible in this study.

### Exercise increases vasomotion in pial arteries

**Fig. 3:**
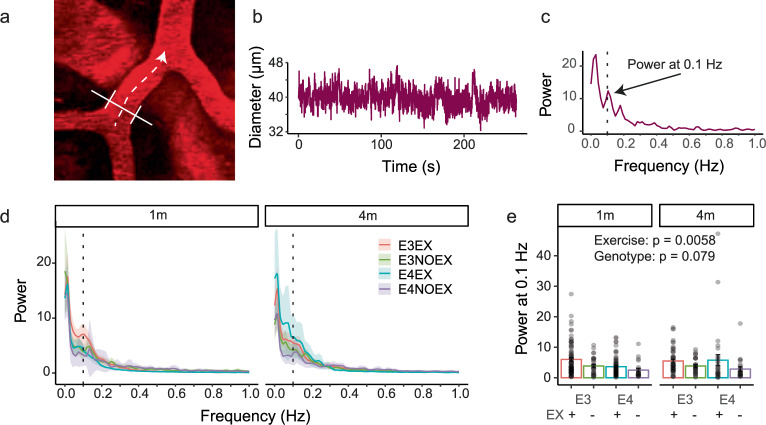
Sedentary mice have decreased vasomotion. **a** Example image of a pial vessel recorded with two-photon microscopy (image from E3EX mouse). Full Width Half Maximum diameter is calculated by scanning perpendicularly to the skeleton of the vessel at each skeleton point. **b** Example of pial diameter trace extracted from two-photon recordings showing spontaneous oscillations in diameter size in the absence of visual stimulation and (**c**) its corresponding power spectrum trace, with peak at 0.1 Hz. **d** Average power spectra of arteriole diameter traces from the experimental groups at the 1- and 4-month time points. Dashed line indicates 0.1 Hz. **e** Exercise increases vasomotion, with active mice showing higher relative power at 0.1 Hz compared to sedentary mice. APOE4 mice show a trend towards lower vasomotion (*p* = 0.08). Dots are average per vessel (22–84 vessels from 4–18 mice). Bars and error bars: mean +/- SEM. For details on *N* and statistical outputs see Supplementary Table 3 in Supplementary Data.

Vasomotion is a low frequency fluctuation in the diameter of large arteries that has been linked to improved oxygen delivery, and our previous work suggests it may be impaired in APOE4-TR mice. Using two-photon microscopy, we recorded diameters of pial vessels in the absence of visual stimuli, after labelling the vascular lumen with 3 kDa Texas Red dextran (Fig. 3a). From the power spectrum of the diameter fluctuations, a peak could be observed at around 0.1 Hz, reflecting vasomotion, as previously reported^29,30^; Fig. 3b, c). In our previous study conducted in mice with free access to exercise wheels, we found a significant effect of APOE4 genotype in reducing vasomotion^12^. Here, we found only a trend level modulation of the size of this peak by APOE genotype, though this reduction in significance was driven by large vasomotion in two vessels from a single active APOE4 mouse at the 4-months time point. Generally, vasomotion was increased in mice with an exercise wheel (Fig. 3d, e) after 1 and 4 months regardless of genotype, suggesting that activity levels modulate vasomotion.

In summary, exercise improved various measures of resting cerebrovascular function, including vasomotion, resting sO2 and capillary density, but clear effects of APOE genotype were absent, except in the likely reduction in vasomotion in APOE4 mice.

### APOE4 sedentary mice have decreased haemodynamic responses to visual stimulation

**Fig. 4:**
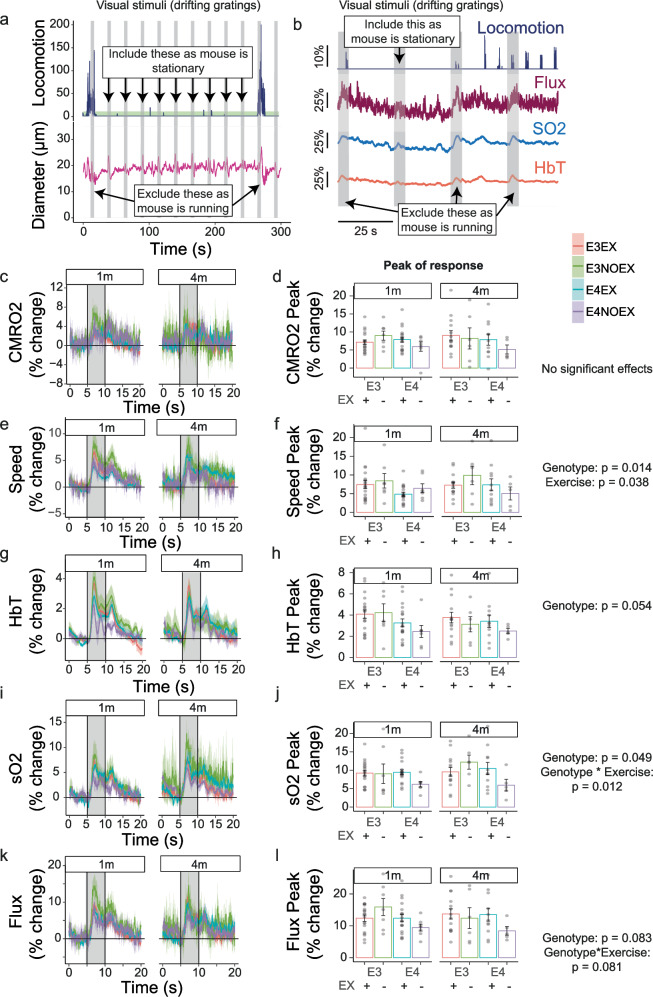
APOE4 mice have lower net haemodynamic responses to neuronal activity, but exercise alters speed of CBF and improves saturated oxygen levels. **a** Example of locomotion trace (top) and vessel diameter recordings (bottom) during visual stimuli presentation (grey bars). Data from stimulus presentations during which the mouse was walking were discarded. **b** Example of net haemodynamic recordings (Flux, sO2, HbT) and locomotion obtained with Oxy-CBF probe during the presentation of visual stimuli (grey bars). **c**, **e**, **g**, **i**, **k** Average traces of haemodynamic responses. **d**, **f**, **h**, **j**, **l** Peak responses (% change from baseline) for each haemodynamic measure. **d** There are no effects of genotype, exercise or experiment duration on CMRO2. **f** Speed of CBF is significantly reduced in APOE4 mice but it is surprisingly higher in sedentary mice. **h** There is a trend towards smaller HbT responses in APOE4 mice. **j** APOE4 genotype and sedentary lifestyle interact to lower sO2 increases during neuronal activity. **l** There is a trend towards a lower CBF flux in APOE4 mice. Dots represent average max peak per animal (*N* mice: 5–19). Bars and error bars: mean +/- SEM. For details on *N* of mice and statistical outputs see Supplementary Table 4 in Supplementary Data.

The brain’s control of its vasculature is highly dynamic, and resting measures do not capture the ability of the system to match blood supply to increased energy demand. To probe whether neurovascular coupling is modulated by activity levels and APOE genotype, we first studied changes in macroscopic haemodynamic responses to visual stimuli (5 s on, 25 s off, repeated 20 times), measured as the maximum peak increase from baseline. To mitigate the confounding effects of locomotion on haemodynamic responses, only stimuli in which the mouse did not run in the 2 s before and 5 s during the stimulus were included in analyses (Fig. 4a, b).

At the macroscopic level, neither genotype, age nor activity level affected the amount of oxygen consumption (CMRO2) during visual stimulation, suggesting that neuronal activation was broadly similar in all conditions (Fig. 4c, d). Across measures of speed, HbT and sO2, there were significant and/or trend-level effects of genotype and exercise and significant and/or trend level interactions between APOE genotype and exercise (Fig. 4e–j), with APOE4 sedentary mice consistently showing the smallest responses. Cerebral blood flux was largely unaltered by genotype, exercise or age of the mice (Fig. 4k, l), although there was a trend towards lower values in APOE4 mice and a trend-level interaction between genotype and exercise as APOE4 mice with a wheel show higher values of flux than mice without a wheel. Supplemental analyses of the area under the curve (AUC) and the time to peak of responses broadly showed a lack of significant effects of genotype, exercise and duration of wheel access, although responses were sometimes slower and trended to be smaller in APOE4 sedentary animals (Supplementary Fig. 3).

### APOE4 genotype and sedentary behaviour reduce vascular responses to increased neuronal activity

**Fig. 5:**
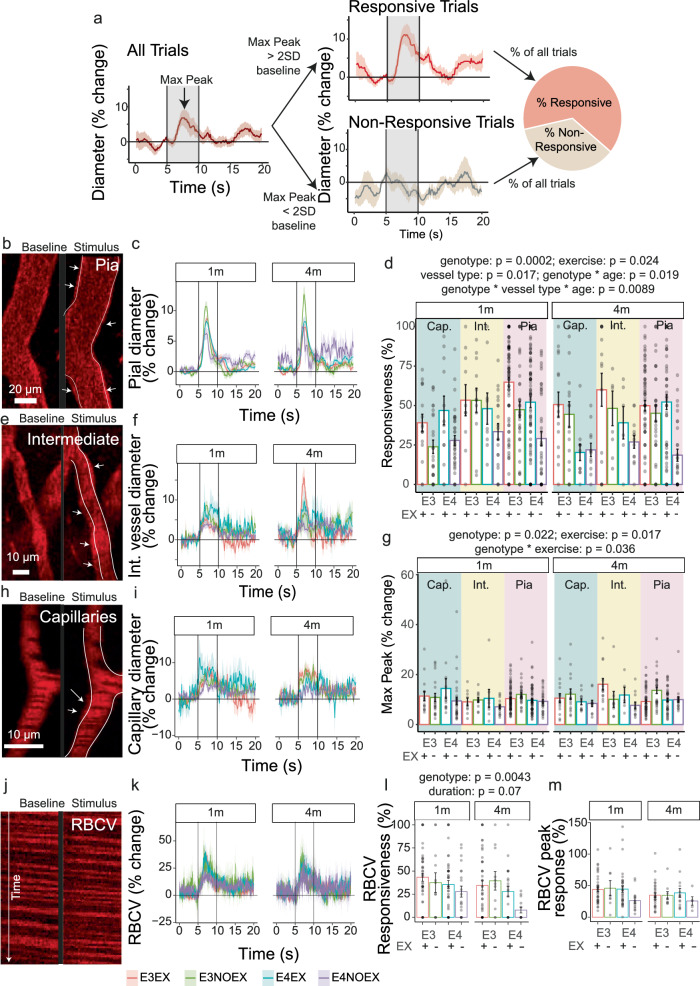
APOE4 vessels respond less reliably to neuronal activity but all except capillary function is improved by exercise. **a** Example of average vessel dilations during the presentation of a visual stimulus (grey area) for one representative arteriole. The diameter trace is normalized to the 1 s preceding presentation of the visual stimulus. The maximum peak dilation during the stimulus is found (Max Peak) and used to determine whether the dilation is larger than 2 standard deviations (SD) from baseline diameter, in which case it is deemed as responsive, or unresponsive if it is less than 2 SD. The number of responsive versus unresponsive trials for each vessel are converted into percentages, to obtain the % of responsive trials (Responsiveness). Example of pial (**b**, from E3EX mouse), intermediate vessels (**e**, from E4EX), capillary (**h**, from E3NOEX mouse) and RBCV (**j**, from E4EX) recordings at baseline (left) and during the presentation of the visual stimulus (right). Lines and arrows indicate where diameter increases in **b**, **e** and **h**. **d** Responsiveness of pial, intermediate vessels, capillaries and RBCV, respectively. APOE genotype, vessel type and exercise affected vessel responsiveness (LMM: genotype * exercise * duration * vessel type; main effect of genotype: *p* = 0.0002; vessel: *p* = 0.02; exercise: *p* = 0.02) (30–95 pial vessels from 4–19 mice; 6–20 intermediate vessels from 3–6 mice; 11–37 capillaries from 3–7 mice). Average response time courses (**c**,**f**,**i**) and peak responses (**g**) for pial, intermediate vessels and capillaries, showing the smallest dilations occur in APOE4 sedentary mice and in capillaries (genotype * duration * vessel type effect: *p* = 0.009) (29–88 pial vessels from 5–19 mice; 5–19 intermediate vessels from 3–6 mice; 10–33 capillaries from 3–7 mice). (**k**,**m**) RBCV response size was not affected by genotype, exercise and age (11–47 capillaries from 3–15 mice). **l** Increases in RBCV were less frequent in APOE4 mice (genotype: *p* = 0.004), with a trend towards less frequent at the later timepoint (duration: *p* = 0.07) (5–39 capillaries from 2–14 mice). Dots represent average max peaks for individual vessels. Bars and error bars: mean +/- SEM. For *N* of mice and statistical outputs see Supplementary Table 5 in Supplementary Data.

To determine what vascular impairments could be causing the changes in macroscopic measures of regional blood flow, oxygen and volume, we studied responses of pial arterioles and parenchymal small arterioles and capillaries to visual stimulation using two-photon microscopy, again excluding any trials in which the mouse was running prior to or during the stimulus. For each vessel, we determined how frequently it responded to the visual stimulus and the size of any responses that did occur (Fig. 5a).

Vessels were classified as capillaries when smaller than 7 µm, pial as superficial vessels with diameter larger than 12 µm, and then all vessels that ranged between 7–12 µm in diameter as intermediate vessels (Fig. 5b, e, h). As we have previously observed^31^, pial arterioles responded more frequently to visual stimulation than capillaries (Fig. 5d, LMM of vessel type * genotype * exercise * experiment duration; main effect of vessel type: *p* = 0.017), whereas intermediate vessels had a responsiveness similar to pial vessels (Fig. 5d). We had previously also found that APOE4 genotype reduced vascular responsiveness in active mice^12^. In this study, vessel responsiveness (Fig. 5d) was again reduced by APOE4 genotype (main effect of genotype: *p* = 0.0002). Exercise increased overall responsiveness (*p* = 0.024). APOE4 mice also showed an overall reduction in vessel responsiveness over time (genotype * duration interaction: *p* = 0.019). Capillaries seemed more sensitive to the interacting influences of APOE genotype with time, as both sedentary and active showed a reduction in capillary responsiveness by 4 months of differential wheel access. Conversely, only sedentary APOE4 mice showed a reduced pial arteriole responsiveness with experiment duration, while responsiveness was preserved in pial arterioles in active mice after 4 months of differential wheel access (genotype * duration * vessel interaction, *p* = 0.0089; Fig. 5d).

When just studying active mice, we previously found no effect of APOE genotype on the size of vascular responses, when they did occur, even though responsiveness was decreased. Here, we found that vessel response size (Fig. 5c, f, g, i) was not different between vessel types but was increased by exercise (LMM of vessel type * genotype * exercise * duration; main effect of exercise: *p* = 0.017) and was overall reduced by APOE4 genotype (*p* = 0.022). The APOE4 effect was impacted by exercise level, with the smallest responses observed in APOE4 sedentary mice (genotype * exercise, *p* = 0.036). Increases in red blood cell velocity (RBCV; Fig. 5j) during visual stimulation reflect dilations in the wider vascular network than a single vessel. These velocity responses were less frequent in APOE4 mice (LMM of genotype * exercise * duration; main effect of genotype: *p* = 0.0043), as seen for vessel dilation responses, and showed a trend-level tendency to be less frequent over time (*p* = 0.074; Fig. 5l). The size of the velocity response did not, however, differ between groups (Fig. 5k, m). The differences observed in the size of vessel responses and response frequency were not due to baseline diameter differences between experimental groups, although APOE3 mice had larger pial vessels than APOE4 mice (Supplementary Fig. 4a). Analysis of AUC of vessel responses to neuronal activity showed that APOE4 sedentary mice had consistently lower responses (*p* = 0.015), whereas APOE4 active mice had preserved responses in pial vessels but a decline in responses in capillaries over time (Supplementary Fig. 4b). Capillaries took longer to reach their maximum dilation than, intermediate vessels and pial vessels (Supplementary Fig. 4c*p* = 0.0075). RBCV AUCs did not differ between groups, but there was a significant delay in RBCV responses to the visual stimulus in APOE4 mice and at the later timepoint. These responses were particularly slow in APOE4 sedentary mice (Supplementary Fig. 4d, e; genotype *p* = 0.044; age *p* = 0.0074; genotype * duration *p* = 0.03; genotype * exercise * duration *p* = 0.0013).

Thus, in these experiments, we found larger impacts of APOE4 genotype on neurovascular responses than previously, with APOE4 reducing both the size and frequency of responses. These responses were increased by exercise, and APOE4-TR mice appeared to be particularly susceptible to either increased time or exercise, with sedentary APOE4-TR mice often showing the weakest neurovascular responses, particularly at the later time point.

### In addition to having neurons that activate less, APOE4 mice have impaired neurovascular coupling

**Fig. 6:**
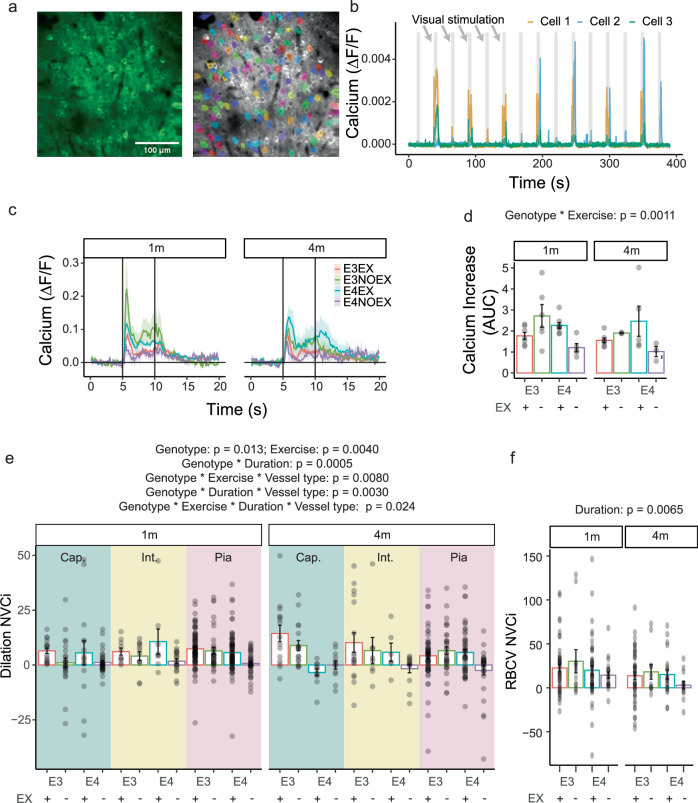
APOE4 sedentary mice have smaller neuronal calcium responses and impaired neurovascular coupling. **a** Example of neuronal calcium recording obtained with two-photon microscopy (left, from E3NOEX mouse) and how ROI masks are created over neuronal soma using Suite2P (right). **b** Extracted traces for 3 cells show an increase in signal during presentation of the stimulus. The trace is cut into 30 s trials that have a 5-second baseline, 5-second visual stimulus and 20-second inter stimulus interval. **c**, **d** Average ΔF/F during visual stimulus shows that APOE4 sedentary mice have smaller neuronal responses to visual stimuli. Dots correspond to average ΔF/F per mouse. (3–8 mice). **e** NVCi is significantly reduced by APOE4 genotype and increased by exercise and exercise duration (LMM for genotype*exercise*vessel type*duration; animal ID as a random factor; genotype: *p* = 0.01; exercise: *p* = 0.004). APOE4 mice have lower NVCi over time (genotype * duration: *p* = 0.0005). Exercise improved NVCi in pial APOE4 vessels but not in APOE4 capillaries, which by the later timepoint were dysfunctional even in active mice (genotype*exercise*vessel type; *p* = 0.008; genotype*duration*vessel type; *p* = 0.003; genotype * exercise * duration * vessel type: *p* = 0.02) (30–95 pial vessels from 5–19 mice; 6–20 intermediate vessels from 3–6 mice; 11–37 capillaries from 3–7 mice). **f** RBCV NVCi reduces over time (LMM for genotype*exercise*vessel type*duration; animal ID as a random factor; duration: *p* = 0.0065) (11–47 capillaries from 3–15 mice). Individual dots correspond to each vessel. Bars and error bars: mean +/- SEM. For details on *N* of mice and statistical outputs see Supplementary Table 6 in Supplementary Data.

Vascular responses could be smaller or less frequent if APOE4 sedentary mice have reduced neuronal activity to visual stimulation, producing a smaller neural drive on signalling processes leading to dilation. However, our previous work in active mice^12^ found that APOE4 genotype did not impact spontaneous neuronal activity but calcium signals were larger in visual cortex neurons during visual stimulation. In this study, in which we incorporate those data with new data from additional active and sedentary mice, spontaneous neuronal activity was unaffected by APOE genotype, exercise or age (Supplementary Fig. 5a–c). Stimulus-driven increases in neuronal calcium levels (Fig. 6a, b) were larger in active APOE4 mice and smaller in APOE4 sedentary mice (Fig. 6c, d; genotype * exercise, *p* = 0.0011). Classifying neurons based on the pattern of their responses (being either activated by the start of the visual stimulus, or the end—when there is a slight decrease in luminance, or activated both the start and the end of the stimulus), revealed that the largest modulation of responses by genotype and exercise occurred in neurons with a broad response pattern, responding to the onset and the offset of the stimulus (cell type * genotype * exercise, *p* = 5.6e–05; Supplementary Fig. 5d–i).

Neuronal calcium signals were reduced in sedentary, but not active, APOE4-TR mice, though not sufficiently to alter CMRO2 (Fig. 4c, d). Thus, the weaker vascular responses in these mice could be due to a reduction in dilatory drive from neurons. To test if vascular responses were appropriately proportional to these smaller neuronal responses, we calculated a neurovascular coupling index (NVCi) by dividing the area under the curve (AUC) of vessel responses during stimulus by the average AUC of neuronal activity during the stimulus in that mouse (Fig. 6e, f). Thus, a reduction in NVCi indicates that there is a smaller vascular response for the same amount of neuronal activity, and therefore that there is a deficit in neurovascular coupling. The NVCi was significantly reduced in APOE4 mice (*p* = 0.013) and increased by exercise (*p* = 0.004). NVCi was particularly reduced in APOE4 mice at the later time point (genotype * duration, *p* = 0.0005). Furthermore, the impact of exercise and duration of wheel access interacted differently with APOE4 genotype across different vessel types (genotype * exercise * vessel: *p* = 0.008; genotype * duration * vessel: *p* = 0.003). For example, exercise improved NVCi in pial APOE4 vessels but not in APOE4 capillaries (Fig. 6e). The NVCi calculated from RBCV responses appeared to follow a similar pattern, but only experiment duration had a statistically significant impact (*p* = 0.0065; Fig. 6f).

Neuronal and neurovascular responses are therefore both negatively impacted by APOE4 genotype and a lack of exercise, with the capillary bed appearing less responsive to the protective effect of exercise.

### Amount of exercise correlates with neurovascular function

**Fig. 7:**
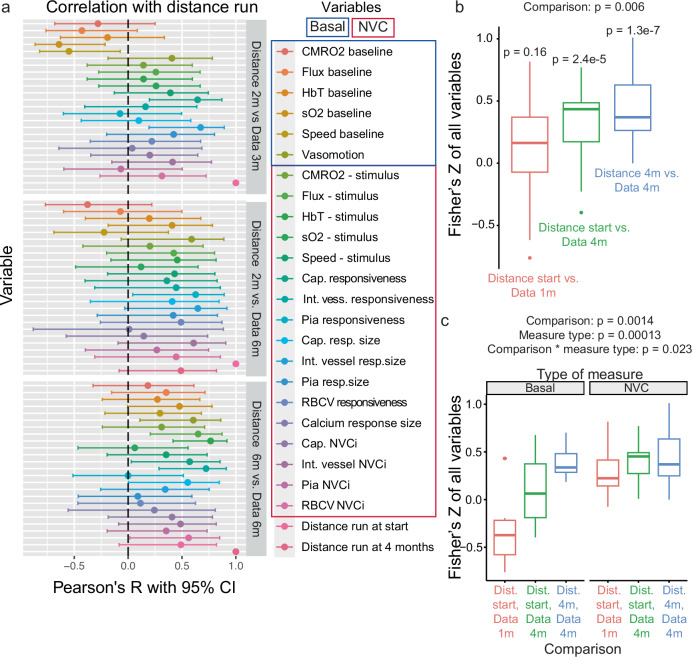
Neurovascular function at 6 months of age correlates with amount of exercise. **a** Data presented in Figs. 2–6 was correlated with the amount of exercise performed by each mouse at the start of the experiment or 4 m of age (5–17 mice). Data shows Pearson’s R with 95% confidence intervals (CI) for the different variables measured. **b** Boxplot of the distribution of correlation coefficients from (**a**) transformed into Fisher’s Z values, across all variables (22 measures). There is an increasing correlation between exercise level and neurovascular function from the start of the experiment to 4 m. *P* values are the result of one sample t tests of Fisher’s Z vs. 0. **c** Box plot of Fisher’s Z combined across 5 measures of basal vascular function or 17 measures of stimulus-induced neurovascular coupling (NVC). NVC measures are overall more correlated with exercise than basal measures, but basal measures become more correlated with exercise over time. *P* values show significant results of ANOVA (condition x measure type) on Fisher’s Z. Full statistical results are in Supplementary Table 7 in Supplementary Data.

While our provision of the wheel resulted in group-wise differences in exercise level (Fig. 1), there were variations in activity level between mice. We therefore interrogated whether the amount of exercise performed by each mouse (normalised across the cohort at that time point) correlated with all the different measures of neurovascular function described above. After controlling for the false discovery rate, no individual correlation was significant (Supplementary Table 6 in Supplementary Data), however overall there appeared to be an increasingly positive correlation between the various features of neurovascular function with time (Fig. 7a). Indeed, overall, the correlation coefficients between all dependent variables after 1 month of differential wheel access and the distance a mouse travelled at the start of the experiment were not significantly different from zero, but the distance travelled at the start of the experiment and after 4 months of wheel exposure were both overall significantly correlated with neurovascular functional measures collected after 4 months of wheel exposure (Fig. 7b). Thus, at the youngest age point, neurovascular function was not related to the amount of exercise carried out, but later on, neurovascular function was related to the current and previous activity levels of the mice. We further probed this increased correlation by subdividing the measures into those of basal neurovascular function (e.g. resting flux, sO2) to measures of neurovascular coupling (e.g. vessel responsiveness to a visual stimulus) (Fig. 7c). Measures of neurovascular coupling were significantly more correlated with exercise than measures of basal neurovascular function, even from an early age. Conversely, the correlation of basal measures of neurovascular function with exercise was low at a young age but increased more over time than did neurovascular coupling measures.

To test whether these effects were affected by APOE genotype or exercise group, we separately calculated the correlations between distance travelled and different haemodynamic variables for each APOE genotype or exercise group (Supplementary Fig. 6a). This reduced the number of data points available to calculate each correlation coefficient, increasing variability. Nevertheless, correlation coefficients between exercise and basal measures of neurovascular function, but not measures of neurovascular coupling were significantly lower in APOE4 mice (Supplementary Fig. 6a, b), suggesting that in APOE3 but not APOE4 mice, basal neurovascular function was greater in mice that did more exercise. Tracking the actual amount of activity performed by each mouse therefore seems to reveal a subtle deficit in APOE4 mice that was not apparent in the group averages plotted in Fig. 2.

Exercise group did not affect correlations between distance travelled and the different measures of neurovascular function, except that the “no exercise” group showed less increase in correlation coefficients over time (Supplementary Fig 6a, c). Potentially, therefore, the enrichment value of having the wheel in the cage may, over time, enhance the correlation between physical activity and neurovascular function in addition to its impact on increasing physical activity.

### Underlying factors driving inter-variable correlations do not show impacts of exercise and genotype

Because several of the variables showed similar correlations with distance travelled, we wondered if underlying factors could be driving similar responses in the different variables, and if these factors might be altered by genotype, exercise and age. A correlation matrix of the different variables studied revealed two clusters of correlated variables, one comprising the baseline haemodynamic responses and the other including all other variables (Supplementary Fig. 7a, b; excluding variables with <80% complete data across animals). To unpick common driving factors underlying these response clusters, we conduced principal component analysis (PCA) separately on the two clusters. Plotting the first two principal components (PC) of the first, baseline cluster did not reveal any clear distinction between the experimental groups (Supplementary Fig. 7c). Only one PC had an eigenvalue over 1, comprising 69% of the total variance (Supplementary Fig. 7d). This PC had similar contributions from all the baseline variables and its value was not significantly different across experimental groups (Supplementary Fig. 7e, f).

In the second cluster of variables, PCA revealed 4 PCs with eigenvalues over 1, accounting for 75% of the total variance (Supplementary Fig. 7g, h). After correcting for multiple comparisons (Bonferroni correction for the number of PCs interrogated), the only significant effect of the experimental manipulations was found in PC2, the main contributing variables to which were vasomotion, pial responsivity and pial NVCi. Experiment duration significantly affected this PC (*p* = 0.01), with APOE genotype trending towards significance (*p* = 0.07; Supplementary Fig. 7i, j).

Thus, PCA did not reveal strong underlying factors driving the pattern of responses observed in individual variables analysed separately, though time and possibly APOE genotype may be affecting a common factor underlying pial physiology.

## Discussion

**Fig. 8:**
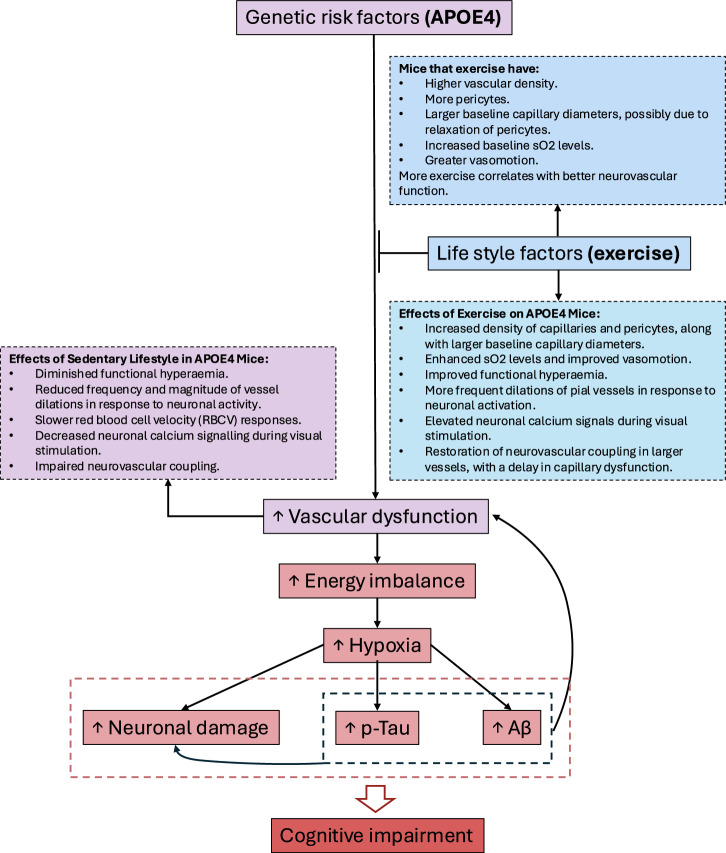
Summary of results—Genetic risk factors (APOE4) and lifestyle factors (degree of physical activity) act synergistically to cause vascular dysfunction. Our results indicate that genetic risk factors, such as the APOE4 genotype, can cause early vascular dysfunction, which is worsened by a sedentary lifestyle (purple panels). Instead, having an active lifestyle can improve measures of vascular function, even when APOE4 genetic risk factors are present (blue panels). Early vascular dysfunction can lead to an increase in energy imbalance, due to poor vascular responses to increased neuronal activity. As a consequence, less nutrients and oxygen are delivered to the brain. Speculatively, this could eventually lead to hypoxia, producing neuronal damage and promoting the accumulation of amyloid-β and p-Tau. These changes in turn can further increase vascular dysfunction and damage neurons (pink panels), setting up a detrimental cycle that culminates with the onset of cognitive symptoms (red panels). Early interventions, such as exercise, may therefore be critical to delay or prevent vascular dysfunction and halt the cascade of events that lead to dementia.

APOE4 is the most common genetic risk factor for Alzheimer’s disease development and a cardiovascular disease risk factor, with many studies suggesting a role for ApoE4 in early vascular dysfunction. To understand how APOE4 increases the risk of developing AD and its effect on neurovascular function, including its interaction with life-style factors, we studied neurovascular function in APOE4-TR and APOE3-TR mice that underwent different levels of voluntary exercise. Our results are summarised in Fig. 8.

APOE4 mice showed neurovascular dysfunction in visual cortex that was particularly evident in mice that were sedentary. In the absence of visual stimulation, there were no differences in neurovascular function due to APOE genotype, in line with our previous findings, but exercise increased capillary and pericyte density, increased baseline capillary diameter, improved vasomotion and increased blood sO2. However, during increases in neuronal activity, APOE4 mice showed a mismatch between neuronal energy demand and vascular responses, with smaller increases in blood flow, HbT, and sO2 levels when neurons were active.

These differences were likely the result of an impairment of vascular responses to neuronal activity. APOE4-TR blood vessels responded less reliably to increases in neuronal energy demand during visual stimulation. When these vessels did respond, the size of their dilations was smaller than in APOE3-TR vessels. However, when these mice exercised, vascular responses to neuronal activity improved—increasing in frequency and size—and this improvement was particularly evident in APOE4 mice.

Neuronal activity was also affected by APOE genotype and exercise level, being lowest in the APOE4 sedentary mice. However, even accounting for this lower level of energy demand, blood vessel responses in APOE4 sedentary mice were disproportionately small (as captured using the NVCi). In arterioles, these weak neurovascular coupling responses of APOE4 mice were improved by exercise, while capillary responses were not.

Finally, measures of neurovascular coupling were correlated with exercise consistently across time, while basal neurovascular function increasingly correlated with exercise as the experiment went on, highlighting the importance of ongoing physical activity for preserving neurovascular function. Interestingly these correlations of basal function with exercise were smaller in APOE4 compared with APOE3 mice. This suggests a subtle deficit in the ability of APOE4 mice to modify their baseline function by increasing their physical activity.

Thus, our results provide evidence that APOE genotype and lifestyle interact to modify neurovascular function. They suggest a deficit in APOE4 carriers in neuronal function, vascular function (as evidenced by reduced vasomotion) and in neurovascular coupling, which could be due to reduced vascular function and/or a reduction in the production of vasodilatory signalling molecules by neurons or astrocytes. Our findings are consistent with observations in human APOE4 carriers showing reduced cerebrovascular reactivity during a CO_2_-inhalation challenge compared to controls^32^ and greater benefits of aerobic exercise on cerebral blood flow^25^. Importantly, our results add mechanistic insight that these changes are due to benefits of physical activity on both neuronal and arterial function, but suggest that capillary level neurovascular coupling and potentially basal neurovascular function may remain impaired in active APOE4 carriers. This interaction between APOE genotype and physical activity may contribute to the variability in cognitive symptom onset and clinical outcomes in human APOE4 carriers, such that those who adhere to a more active lifestyle might delay or prevent the vascular dysfunction that precedes dementia. Our results also provide a potential explanation for the variability of results in the mouse literature as to the impact of APOE genotype on neuronal and vascular function: previous studies found a dramatic reduction in CBF response to whisker stimulation in APOE4-TR mice of the same age as the ones we used for this study^9,10,33^, while we previously found only a very mild neurovascular phenotype in APOE4-TR mice^12^. However, the studies showing large deficits in neurovascular function were conducted in acute surgical preparations and/or anaesthetized mice, while our previous study was conducted in awake mice that had fully recovered from the cranial window surgery and had ad libitum access to an exercise wheel. Our new data reveal that this mild neurovascular deficit in APOE4-TR mice is worsened by lack of physical activity. Thus, together, these results suggest that APOE4 genotype does not result necessarily in a devastating loss of neurovascular function, but puts APOE4-TR mice, and human APOE4 carriers, at greater risk of neurovascular damage during conditions of additional stress. This is consistent with human data showing cognition in APOE4 carriers to be more sensitive to lifestyle differences including physical activity^34^ and anaesthesia^13,14,35^, and evidence that APOE4 genotype mediates increased cell damage in response to stressors such as inflammation^15^.

It is noteworthy that, in this study, the largest reductions in both neuronal and vascular function in APOE4-TR mice occurred in sedentary mice during conditions of increased energy demand (i.e. during visual stimulation when neuronal activity was increased), after a long differential exposure to exercise, and it may be that this increased neuronal activity represents an additional challenge with which APOE4-dependent processes are less able to cope. Because neuronal activity was reduced in APOE4 mice, but also the frequency and relative size of neurovascular coupling responses were reduced, this suggests two partially separable deficits—the reduction in neuronal activity, and an alteration in the vascular response to neuronal activity (or in production of molecules that signal the increase in activity to the vasculature). This reduction in neuronal calcium signals during visual stimulation, but not at rest, is consistent with the larger reduction in glucose uptake in APOE4-TR compared to APOE3-TR mice that occurs during a cognitive challenge compared to during rest^36^. However, while APOE4 is generally found to reduce both glucose and oxygen metabolism^37^, older APOE4 carriers and APOE4-TR mice have been reported to show signs of neuronal hyperactivity and increased energy metabolism, with the largest effects in the entorhinal cortex^38–40^. The reduced neurovascular coupling we observed even at a young age would suggest the vasculature of APOE4 carriers will not be able to match increased blood flow to this increasing demand. It will be of future interest to determine how such an evolving mismatch contributes to ongoing, accelerating dysfunction in the ageing brain.

Exercise is well known to improve cardiovascular function, and previous studies found that exercise increased cerebrovascular reactivity of arterioles in healthy animals and in mouse models of disease^41,42^. Our findings extend this, showing that pial arteries are more sensitive than the capillary bed to the impact of exercise, as well as that the beneficial effects of exercise may be greatest for APOE4 carriers. The mechanism underlying these changes remains unknown, and several possibilities are suggested by the existing literature:

Exercise can directly reduce plasma levels of the potent vasoconstrictor endothelin-1 (ET-1) by increasing pulsatile-induced shear stress via increased blood flow^43^ and by reducing the synthesis of its precursor, big-ET^44^. In addition to altering ET-1 levels directly, exercise may act via altering levels of ROS. Exercise can reduce superoxide production by reducing the activity of NADPH oxidase and increasing the activity of the superoxide dismutases SOD1 and SOD3^45^. ET-1 production is promoted by ROS, which are found at higher levels in APOE4 mice^9,15^ and ROS-stimulated ET-1 production constricts capillary pericytes^28^, thus an exercise-mediated reduction in ROS could reduce ET-1 production and reduce capillary pericyte constriction, which will promote blood flow^46^. Indeed, reducing levels of superoxide can improve blood flow, as the superoxide scavenger MnTBAP ameliorated the large decrease in CBF observed in acute surgical preparations of APOE4 mice^9^.

Thus, exercise might reverse the vasoconstriction and reduced vasoreactivity caused by APOE4-induced ROS accumulation by reducing the synthesis of ET-1 and promoting the metabolism of ROS. ROS levels increase with age^47^, which could underlie the increased impact of exercise on neurovascular function with increased age of the mice. As ROS generation within the parenchyma is also associated with dysfunctional neuronal signalling^48^ and this can be mitigated by exercise^49^, such a pathway may also potentially underlie the influence of exercise and APOE genotype on neuronal activity.

In addition to reducing the production of the vasoconstrictive ET-1, exercise may improve vasodilation by increasing levels of available nitric oxide (NO). NO production from endothelial nitric oxide synthase (eNOS) is increased by exercise, acutely due to increased endothelial shear, and chronically during prolonged exercise training^50^. NO production from neuronal nitric oxide synthase (nNOS) is stimulated by intracellular calcium increases during neuronal activity^51^, contributing to neurovascular coupling^52^, therefore the increased neuronal activity in active APOE4 mice likely generates more NO than the smaller calcium signals in sedentary APOE4 mice. nNOS is largely expressed in interneurons in neocortex, the activity of which is generally increased in parallel with increases in excitatory neuronal activity like the signals we measured here. NO bioavailability is reduced by its rapid reaction with ROS, levels of which, as discussed above, are increased by APOE4 genotype and reduced by exercise. Thus, NO bioavailability is likely reduced in APOE4 sedentary mice via effects on both its synthesis and breakdown. An involvement of altered NO levels in the genotype and exercise-mediated alterations in neurovascular function may explain the greater impact of exercise on arteries than capillaries, as NO has been shown to have a bigger vasodilatory effect on arteries than capillaries^53^.

Neurovascular coupling may also be affected by alterations in astrocyte communication with blood vessels. APOE4 mice have been reported to have reduced levels of aquaporin 4 (AQP4) in astrocytic end feet. AQP4 is important for maintenance of vascular tone and neurovascular coupling^54^. Exercise increases the levels of perivascular AQP4, as seen in a mouse model of vascular dementia that was subject to exercise^55^. Thus, exercise might recover impaired vascular responses to neuronal function by promoting the presence of perivascular AQP4 channels.

Finally, the CypA/MMP9 pathway has been linked to pericyte dysfunction and increased BBB permeability in APOE4 carriers^6^ and APOE4-TR mice^10^. This pathway could potentially impact contractile function by increasing vascular stiffness^56^, but it is not clear how it is modified by exercise. Levels of MMP9 mRNA are increased by exercise, for example^57^, but its activity may be reduced by prolonged exercise training^58^.

A plausible hypothesis is therefore that: 1) increased ROS in sedentary APOE4-TR mouse neurons and blood vessels produce increased ET-1 mediated constriction across the vascular bed, reduced neurovascular coupling due to reduced AQP4 in astrocyte end feet, and reduced neuronal activity, and 2) exercise mitigates these deficiencies by reducing ROS, thereby reducing ET-1 synthesis and increasing NO bioavailability and by increasing AQP4 levels in astrocytes. Testing this hypothesis and unpicking the relative contribution of these different pathways is an important topic for future investigation, and could suggest therapeutic targets to improve neurovascular function in both sedentary and active APOE4 carriers.

In addition to changes in neurovascular function, we also observed that exercise, but not genotype, increased capillary density and diameter near pericytes, with a potential increase in pericyte density. This corroborates previous work showing that exercise increases capillary density through angiogenesis^59^. This effect may be mediated by exercise-induced increases in lactate activating hydroxycarboxylic acid receptor 1 (HCAR1) receptors located on pial fibroblast-like cells and capillary pericytes. Activation of HCAR1 leads to elevated vascular endothelial growth factor (VEGF) A protein levels, ultimately promoting capillary angiogenesis^21^.

APOE4 mice have also been shown to exhibit reduced VEGF levels^60^, which could contribute to the reduced capillary density observed in some studies^8–10^. However, we did not observe a reduction in capillary density in APOE4 mice. The reason for this discrepancy is unclear. The previous studies included mice that were significantly older than the mice in our study^8^, but also those of an equivalent age^9^. Methodological differences may contribute: For example, we quantified vascular density by filling the vasculature with a fluorescent gelatine, while other studies immunolabelled endothelial markers, so perhaps APOE4 reduces marker expression without affecting vessel perfusion. Whatever the reason, our data suggest that it is not necessarily the case that APOE4 expression leads to a reduction in vascular density. Indeed, no such reduction has, to our knowledge, been observed in healthy humans. Instead, APOE4-associated reductions in vascular density have only been documented in APOE4-positive Alzheimer’s disease patients (e.g^11^.), where the effects could be due to an interaction of APOE4 with beta amyloid, tau or inflammatory pathology.

Together these reports suggest that APOE4 genotype can sometimes promote a reduction in vascular density, but this effect may become more apparent in the context of additional pathology. When this is the case, exercise-induced VEGFA production may help counteract these genotype effects, supporting healthy capillary density, but this would need to be specifically tested in new experiments.

Adding a wheel to the cage manipulates the physical activity of the mice, but also adds environmental enrichment. Environmental enrichment is known to improve brain and cognitive function models of ageing and disease and, like exercise, can increase NO levels^61–63^. Dissecting the relative contributions of increased physical activity and cognitive stimulation of environmental enrichment is challenging and requires tracking home cage activity as we have done here. Because activity level was correlated with both basal and neurovascular coupling measures, physical activity is likely a major driver of the alterations in neurovascular function that we observe. However, in our experiments, the “no wheel” group showed a smaller increase over time in the correlation between physical activity and neurovascular function than the “wheel” group. This may mean that cognitive stimulation provided by the wheel modifies the relationship between physical activity and neurovascular function. Future experiments with larger group sizes to better capture correlations in subgroups are needed to test this hypothesis.

Altogether, our results show a consistent pathophysiological effect of APOE4 on neurovascular function during times of greater neuronal activity, which is worsened by a sedentary lifestyle. These effects are seen in reductions in net hemodynamic measures, in individual arteries’ and capillaries’ dilations to neuronal activity, and in the activity of neurons themselves in APOE4-TR mice. Importantly, we found that exercise can improve most, but not all, of these measures, providing an explanation for the previous conflicting results reported in the literature, and suggesting that physical exercise might protect human APOE4 carriers against worsening neurovascular dysfunction and the emergence of dementia (Fig. 8).

## Methods

### Animals

Procedures involving animal use were conducted in accordance with the UK Animals (Scientific Procedures) Act 1986 with the approval of the University of Sussex Animal Welfare and Ethical Review Board. We have complied with all relevant ethical regulations for animal use. Breeding was carried out in-house for APOE4-TR mice (B6.129P2-Apoetm3(APOE*4)Mae (MGI:2158398) at N8 backcross to C57BL/6) and for APOE3-TR mice (B6.129P2-Apoetm2(APOE*3)Mae (MGI:2157240) at N8 backcross to C57BL/6) from founders provided by N. Maeda (UNC School of Medicine, USA). In these mice, the exons 2–4 of murine APOE were targeted for replacement with exons 2–4 of human APOE, resulting in the production of human ApoE^64,65^. APOE3-TR mice and APOE4-TR mice were bred either with mice expressing GCaMP6f under the control of Thy1-promoter (C57BL/6J-Tg(Thy1-GCaMP6f)GP5.5Dkim/J (MGI:5523959) available from The Jackson Laboratory, on C57BL/6 J background)^66^ or with mice expressing DsRed under the control of the NG2 promoter (Tg(Cspg4-DsRed.T1)1Akik/J (MGI:3800939), available from The Jackson Laboratory, on C57BL/6 J background)^67^.

### Surgery

8–12 week old APOExThy1-GCaMP6f mice underwent a cranial window surgery over the visual cortex (V1) as previously described^68^. Briefly, mice were anaesthetised using isoflurane (maintained between 1 and 2%) while secured on a stereotaxic frame. Subcutaneously, mice were given 2.4 μl/g of dexamethasone (2 mg/ml) to reduce inflammation, 400 μl of saline to reduce dehydration and 1.6 μl/g of buprenorphine (0.3 mg/ml, diluted 1:10 with saline) to reduce post-operative pain. The scalp and underlying periosteum were removed across the dorsal skull surface. A custom-made stainless-steel head plate was fixed on the skull using black dental cement (Unifast Powder mixed with black ink (1:15 w/w) and Unifast Liquid) that was placed all over the skull surface with exception of the area overlying V1. A 3 mm craniotomy over V1 was performed using a dental drill (Fine Science Tools, burr size 007), and a 3 mm glass coverslip bonded with a 5 mm glass coverslip (Harvard Apparatus) was placed into the craniotomy and secured using Vetbond and dental cement. Following surgery, mice recovered in a heat box (37°C) and then were singly housed in a recovery cage. Post-surgery meloxicam (200 μl, 1.5 mg/ml) was administered orally in the food for 3 days for additional pain relief during recovery. Prior to imaging and following a post-surgery recovery period of at least 2 weeks, mice were gradually habituated to head fixation on a polystyrene cylinder over 4–5 sessions of increasing duration.

### Exercise manipulation

Post-surgery, some of the mice were provided with an exercise wheel in the cage, whereas for others no wheel was added. Initial randomisation of wheel allocation was achieved by blindly selecting a wheel or no wheel condition for the first mouse in a litter of a given sex to undergo surgery. The next mouse from that litter and sex was then provided with the opposite condition to match littermates across conditions. If there were more mice of a given litter and sex used for the experiment then the experimental condition was again randomly selected for the next mouse, which was then counterbalanced by the following mouse, and so on. Later in the experiment, if there were fewer mice in a given experimental group, then mice were prioritised for the conditions where the sample size was smaller.

### Recording mouse activity from the home cage

To track the activity levels of the mice, a USB Camera Module with IR LED for day night video surveillance (ELP USB Webcam, Ailipu Technology Co., Ltd.) was placed in a custom-made 3D printed support that attached to the top of the home cage and was connected to a computer through a USB cable. A custom code written using Bonsai.rx was used to control the cameras, acquiring 4-hour long videos (240 × 360 pixels) for 72–120 h consecutively at a rate of 30 frames per second. A dark marker was placed at the edge of the running wheel, to enable tracking of the number of wheel rotations. Materials and methods for this recording set up are freely available on GitHub: https://github.com/Sussex-Neuroscience/rodent-tracking. Mice were allowed to recover from surgery for at least 2 weeks before their activity levels were recorded. Activity recordings were conducted on the same mice, whenever possible, at 2-months and 6-months of age, i.e. at the start and after 4 months of differential wheel access (*N* = 1 mouse died before the second time point and *N* = 2 mice had to skip the first recording time point because of COVID-19 restrictions).

### Vascular and pericyte density post-mortem

2-month-old surgery-naïve APOE3 and APOE4 mice crossed with NG2-DsRed mice were housed either with or without the wheel in the home cage for 1–1.5 month before being transcardially perfused as described previously^68^. Briefly, after mice were injected with an overdose of pentobarbital (1.5% sodium pentobarbital at a dose of 0.06 ml per 10 g), the heart was exposed and transcardially perfused with ice-cold PBS for 5 min, followed by 15 mins of ice-cold 4% paraformaldehyde (PFA), 5 min of PBS at 36 degrees and finally 3 min of 5% gelatine containing 0.2% fluorescein isothiocyanate (FITC)-conjugated albumin at 36 degrees. Gelatine was set by chilling the mouse on ice for 30 min. Brains were then collected and post-fixed in 4% PFA for 24 h before being sliced into 200 µm thick slices using a vibratome (Vibratome® and Leica VT1200S), that were whole-mounted on a microscope slide (SuperFrost, Thermo Scientific) and covered with Vectashield Antifade Mounting Medium (Fisher Scientific) and glass coverslips (Menzel 24 × 60 mm 0.13–0. 16 mm, Thermo Scientific). Slices were imaged using a Leica SP8 TCS confocal microscope (Leica Microsystems) using a 20x air objective (HC PL APO CS2 20X/0.75, Leica Microsystems). Continuous wave lasers with excitation wavelength of 488 nm and 555 nm were used to collect 1024 × 1024 pixel matrix Z-stacks of FITC-labelled brain vasculature and NG2-DsRed labelled pericytes from V1, with a line average of 4 and pixel size of 0.45 × 0.45 × 1 μm.

### In vivo recordings

For in vivo recordings of neurovascular function, awake mice were head-fixed over a rotating cylinder that allowed for voluntary locomotion. The movement of the cylinder was recorded using a rotary encoder (Kuebler, Germany). For each measure, recordings were collected during “baseline”, when the mice were in the dark and not walking, or during the intermittent presentation of a 5-second drifting grating visual stimulus (315°, 2 Hz full screen stimulus, with alternating spatial frequency trials of either 0.04 or 0.2 cycles per degree) on two computer screens (Asus) placed about 20 cm away from the mouse. Each stimulus was followed by 25 s of a grey screen (rest) to allow responses to return to baseline, after which another 5 s stimulus was presented. For each recording, 20 visual stimulation trials were presented. The luminance of the screens during the drifting gratings was not matched to the luminance in the absence of the visual stimuli, being brighter during the stimulus (0.85 µW) than at rest (0.54 µW).

### Oxy-CBF probe

A combined laser Doppler Flowmetry/ haemoglobin spectroscopy probe (Oxy-CBF probe; VMS OXY/LDF Moor instruments) was used to record net haemodynamic measures across a tissue volume of around 500 × 500 × 1000 μm^69^ at a frequency of 40 Hz from the visual cortex of the mice. Measures of blood flow (CBF), speed of CBF, oxy- (Hbo), deoxy- (Hbd) and total haemoglobin (Hbt), saturated oxygen levels (sO2) and cerebral metabolic rate of oxygen consumption (CMRO2) were recorded both at baseline and during the presentation of a visual stimulus.

CMRO2 was calculated as in ref. ^70^ (Equation 1).

1 $${CMRO}2={CBF}\times \frac{{Hbd}}{{Hbt}}$$

### Two-photon microscopy

A two-photon microscope (Scientifica) and a mode-locked Ti-sapphire laser (Coherent) were used to visualise vessels and neurons in the visual cortex of APOE-TR mice expressing GCaMP6f under the control of the Thy1 promoter^66^. Visualisation of cortical vasculature was possible due to subcutaneous injection of 100 μl 2.5% Texas Red dextran 3KDa (Sigma Aldrich) prior the beginning of the imaging session. Recordings were taken with a 16x water immersion objective (CFI LWD Plan Fluorite, 3.0 mm working distance, Nikon) at an excitation wavelength of 940 nm. Blood vessel and neuronal recordings were taken over 256 × 256 pixels, at a frame rate of 7.63 Hz, and a mean pixel size of 0.23 μm (range: 0.15–0.63 μm). High speed line scans were collected to allow measurement of red blood cell velocity (RBCV) at a mean rate of 1092 Hz (range: 413–2959 Hz), with a mean pixel size of 0.20 μm (range: 0.15–0.46 μm). To prevent light artefacts caused by the appearance of the visual stimulus on the screens, the objective was shielded with a rubber black tape. SciScan software (Scientifica) was used to collect recordings. Thy1-GCaMP6f mice show variable expression levels of GCaMP6f in V1^71^ so collection of neuronal calcium data was not possible in all mice (26/52 mice lacked sufficient GCaMP6f expression to collect calcium imaging data).

### Experimental cohorts

This study used three cohorts of mice. Each cohort contained mice of both sexes. Analysis of sex-dependent effects (LMM with sex as a fixed factor) found only a few significant sex effects, which lacked a clear pattern. It is of note that the number of mice in some of the experimental groups was low when stratifying by sex, thus the analysis of sex effects was very underpowered. We nonetheless conducted these analyses in case any clear patterns of sex difference emerged that would warrant further follow up and in case future studies might benefit from this information. A summary of observed sex effects (where present) can be found in Supplementary Table 8 and all statistical outputs are in Supplementary Table S7, both in in Supplementary Data.

The first cohort of mice (APOE-TR x NG2-Dsred) had access to an exercise wheel from 2 months of age (4 APOE3-TR mice (2 females, 2 males) and 3 APOE4-TR mice 2 female, 1 male) or no exercise wheel (5 APOE3-TR mice (2 females, 3 males) and 7 APOE4-TR mice 4 female, 3 male), and were transcardially perfused after 1–1.5 month to collect regional vascular density and pericyte measurements. The data from mice with access to an exercise wheel have been previously published^12^.

The second cohort of mice (APOE-TR x Thy1-GCaMP6f) underwent surgery to implant a cranial window over visual cortex at 8–12 weeks after which they either had access to an exercise wheel (4 APOE3-TR mice (2 females, 2 males) and 4 APOE4-TR mice 2 female, 2 males) or no exercise wheel (6 APOE3-TR mice (3 females, 3 males) and 6 APOE4-TR mice 3 female, 3 males). Physical activity levels were recorded in the home cage of these mice at 2 or 6 months of age, and vascular, neuronal and haemodynamic recordings were collected from the visual cortex in the in vivo experimental setup at 3 and 6 months of age (i.e. after 1 or 4 months of differential wheel access). Periods of exercise and in vivo imaging time points were chosen to match the age at which recordings were conducted in our previous study^12^. Voluntary exercise was recorded at the start of the experiment at 2 months of age to capture the highest activity levels performed by each mouse. This time point was chosen because mice are more active at 2 months than at older ages and physical exercise at 2 months have the greatest changes in transcription of genes involved in cerebrovascular remodelling^27,62^.

The third cohort of mice (APOE-TR x Thy1-GCaMP6f;15 APOE3-TR mice (7 females, 8 males) and 15 APOE4-TR mice (8 female, 7 males)) underwent surgery to implant a cranial window over visual cortex at 9–12 weeks of age after which they were provided with an exercise wheel. In vivo vascular, neuronal, and haemodynamic recordings were taken from the visual cortex at 3 and 6 months of age (i.e. 1 or 4 months of wheel access). Data from these mice has been previously published^12^. In this study, oxy-CBF recordings, pial vessel recordings, RBCV recordings and calcium recordings from these mice have been combined with the data from the second cohort of mice.

It was not always possible to collect all measures of vascular function, neuronal activity and exercise levels from the same mouse, so details on the number of mice and number of observations per group for each measure can be found in the supplementary statistical information.

### Data analysis

All code used to extract data from collected images is published on archived GitHub repositories linked in the data availability section. All analysis was conducted using automated analysis and/or with the experimenter blind to the experimental condition and mouse genotype.

### Analysis of mouse exercise

Animal activity was extracted using DeepLabCut (DLC). DLC is a software package for animal pose estimation that allows to create annotated training sets, train robust feature detectors and utilise them to analyse behavioural videos^72,73^. The multi-animal version of DLC was used as it allows to label both the mouse and the wheel rotation marker. To train the network, 20 frames/video were manually extracted from a subset of the 10 min videos, including videos from mouse cages containing and not containing an exercise wheel. To optimise the accuracy of the model training, we included videos that showed the mouse engaging with different behaviours, in diverse environments and lighting conditions. The extracted frames were manually labelled for mouse body parts (head, left headplate, right headplate, tail) and for the dark marker on the wheel (inner marker and outer marker). Once all frames were labelled, the network was trained using the Google Collab version of DLC. Training lasted for 50,000 iterations, as recommended by the creators of DLC. Following completion of training, videos were uploaded to Google Drive and analysed using the trained network. A.csv file containing the X and Y coordinates of each label in each frame was outputted and saved in Google Drive for each of the analysed videos.

The extracted.csv files containing XY positions of labels were then analysed using custom written scripts in R. As each coordinate labelled by DLC comes with a probability value between 0 and 1 of the label being accurate, we set a threshold of 1 to filter our observations so that only the most accurate tracked locations were included. We then calculated the change in centroid position of the mouse and wheel rotation marker for each frame and summed these over a 10-minute interval to obtain a value for the distance travelled in 10 min. The top and bottom 1% of the distribution were filtered out to exclude extreme outliers (which occurred when there was a failure to classify a frame). From the remaining data, we calculated the total movement of each mouse over 24 h.

### Analysis of post-mortem vasculature and pericyte density

Vascular density was calculated using a custom-written ImageJ macro written by Dr. Devin Clarke. The macro averages across imaging planes, allowing removal of noise. The stacks are then automatically thresholded using the Default, Huang, Huang2 or Li thresholds, depending on which the (blinded) observer judged provided the best segmentation. A 3D skeleton was created along the centre of the vascular lumen, skeleton co-ordinates and branch points were determined and a distance map of the vasculature was created. From the output of the “Analyse skeleton” plugin, total vessel length was calculated and employed to calculate the vessel density. Vessel radius was calculated from the distance map value at each skeleton point. Analysis was performed when blinded to brain region and experimental condition. Pericyte cell soma location was manually marked using the multi-selection tool in ImageJ and vessel diameter as a function of distance from pericyte soma and pericyte density was calculated using a MATLAB code written by Dr. Dori Grijseels.

### Extraction of in vivo data

For recordings of vascular diameter and red blood cell velocity (RBCV), brightness and contrast were optimised in ImageJ (Fiji) using the built in Brightness/Contrast function and the Stack Contrast Adjustment plugin was used to ensure all frames in the same recording had the same contrast. For vessel recordings, all frames were registered using the Template Matching plugin in ImageJ (Fiji) to control for any motion artefacts.

A custom written MATLAB code was used to determine the vessel diameter from the average full width half maximum (FWHM) of the fluorescence intensity profile calculated over a running window perpendicular to the vessel’s axis, whereas RBCV was extracted from high-speed line scans using a radon transform modified from a previously published code^74^.

### Analysis of baseline measurements (no visual stimulation)

For haemodynamic recordings in the absence of visual stimulation, periods of no locomotion lasting at least 10 s were identified and an average value calculated across these rest periods per imaging session for each parameter. At each time point, data points were normalised to the average value of APOE3 exercise mice to remove any variability due potential changes in sensitivity of the Oxy-CBF probe over time between data collection for cohorts 2 and 3. The cohorts did not show any significant differences before normalisation.

Neuronal recordings at baseline were analysed for periods of no locomotion that lasted at least 10 s. Peaks per minute and size of the calcium peaks were determined for each rest period and again averaged per imaging session.

### Analysis of vasomotion

Vasomotion was estimated from Welch’s power spectral density of the diameter changes across the whole baseline recording session (i.e. when there was no visual stimulation, but running bouts were included) as we previously showed that running does not correlate with increased vasomotion^12^. Using the built-in MATLAB functions “detrend” and “pwelch”, traces were first detrended and then discrete Fourier transforms were conducted. Because the resulting traces from the Fourier transform have different sizes, linear interpolation was used to ensure all traces were the same length. On these traces, we then discarded any value that was higher than 1 Hz. We then estimated the degree of vasomotion from the power density of each trace at 0.1 Hz^30^.

### Analysis of neurovascular responses to visual stimulation

Stimulus-induced haemodynamic measurements and two-photon vessel and neuronal recordings were cut into 30 second-long trials; with 5 s baseline (prior to stimulus presentation), 5 s of visual stimulus, and a post stimulus 20 s interval. Only the trials in which the mouse did not run in the 2 s prior and during the stimulus presentation were considered in the analysis to exclude the confound of locomotion. Trials were averaged to yield a mean response per vessel (in vivo vessel recordings) or animal (Oxy-CBF and neuronal recordings). Responses for single vessels were then classified as responsive or non-responsive to the visual stimulus by comparing the peak height with the baseline variability, calculated from the standard deviation of the values during the 5 s baseline period. If the peak signal during the 5 s of the visual stimulus was greater than 2 standard deviations of the baseline, the signal was deemed to be responsive. Based on the number of responsive trials per vessel, a response rate was calculated (% responsiveness). For neuronal recordings, traces from individual cells were classified as belonging to ON cells when the signal increased during the visual stimulus, OFF cells when the signal increased after the stimulus (i.e to the drop in luminance), or BOTH cells when the signal increased both during and after stimulus, and the average AUC per response category per animal was calculated. To link vascular and neuronal function, neurovascular coupling indices (NVCi) were calculated by dividing individual AUC of each vessel by the average neuronal AUC for each genotype (as calcium data was not available for all mice due to inconsistent GCaMP6f expression between mice).

### Statistics and reproducibility

Graphing and statistical analyses were performed using RStudio with data and code used to produce each figure are linked from the data availability statement. Graphing used the *ggplot2* package^75^. For each graph, data points represent either average vessel data or averages per animal as stated in the legend. The summary bars show mean values across the whole experimental group +/- SEM, with standard deviations reported in the Supplementary Information. Where possible, data was analysed with linear mixed models (LMM) using the *lmer* function from the *lme4* package^76^, with random effects as specified in the text and/or in Supplementary Tables 1:7 in Supplementary Data. A two-way ANOVA was conducted on each LMM to determine the significance of any fixed factors (genotype, exercise, age for in vivo analyses and also brain region for ex vivo analyses), while accounting for random factors (animal ID and cohort). Two-tailed *p* values lower than 0.05 were considered as significant, whereas values lower than 0.1 were classified as trending towards significance. Exact *p* values are provided in the figures where they are significant and all test statistics and full *p* values are in Supplementary Tables 1:7 in Supplementary Data.

Correlation analysis used the *cor.test* R function from the *stats* package^77^ to calculate correlation coefficients and p values, and the *ci_cor* function from the *confintr* package^78^ to calculate confidence intervals. Correlation coefficients were transformed into Fisher’s Z values using equation 2, below, where *r* is Pearson’s R and *Z* is Fisher’s Z.

2 $$Z=\frac{1}{2}{{\mathrm{ln}}}\left(\frac{1+r}{1-r}\right)$$

To conduct the principal component analysis (PCA), the degree of missing data was first quantified across the different variables using *aggr* in the *VIM* package^79^ and any variables with more than 20% of the value missing were excluded (e.g. where exercise was not recorded). Missing data in the remaining variables was imputed using the *mice* package (Multivariate Imputation by Chained Equation^80^). Correlation matrices were plotted using the *corrplot* package and PCA performed and visualised using the *prcomp, screeplot* and *biplot* functions in the *stats* package, as well as the *plot3D* package^81^, for 3D plots. Within each analysis, principal components (PCs) with eigenvalues over 1 were retained and the values of these PCs compared across groups using a LMM. The *factoextra* package^82^ was used to extract the contributions of different variables to the different PCs.

### Reporting summary

Further information on research design is available in the Nature Portfolio Reporting Summary linked to this article.

## Supplementary information

Supplementary Information

Supplementary Data

Description of Additional Supplementary Files

Reporting Summary

Peer Review file

## Data Availability

Processed data used to create each figure is available at Figshare, 10.25377/sussex.25436869. Raw images are available from the authors on request.

## Code availability

Code used to extract data from images is available at 10.5281/zenodo.14755861, archived from our GitHub site https://github.com/BrainEnergyLab/VascularImagingAnalysis. Code used to generate each figure from these data is available at Figshare, 10.25377/sussex.25436869.
