# Supplementary material for: APOE4 and sedentary lifestyle synergistically impair neurovascular function in the visual cortex of awake mice
Source: Commun Biol. 2025 Jan 29;8:144. doi: 10.1038/s42003-025-07585-z (PMC11779976; doi:10.1038/s42003-025-07585-z)
Supplement: Supplementary file 1 — Supplementary Information [file 42003_2025_7585_MOESM1_ESM.pdf]

Supplementary Information: APOE4 and sedentary lifestyle synergistically impair neurovascular function in the visual cortex of awake mice

Silvia Anderle[1,2], Orla Bonnar[1,3], Joseph Henderson[1,4], Kira Shaw[1], Andre M Chagas[1], Letitia McMullan[1], Alexandra Webber[1], Kirsty McGowan[1], Sarah L. King,[1] Catherine N. Hall[1]

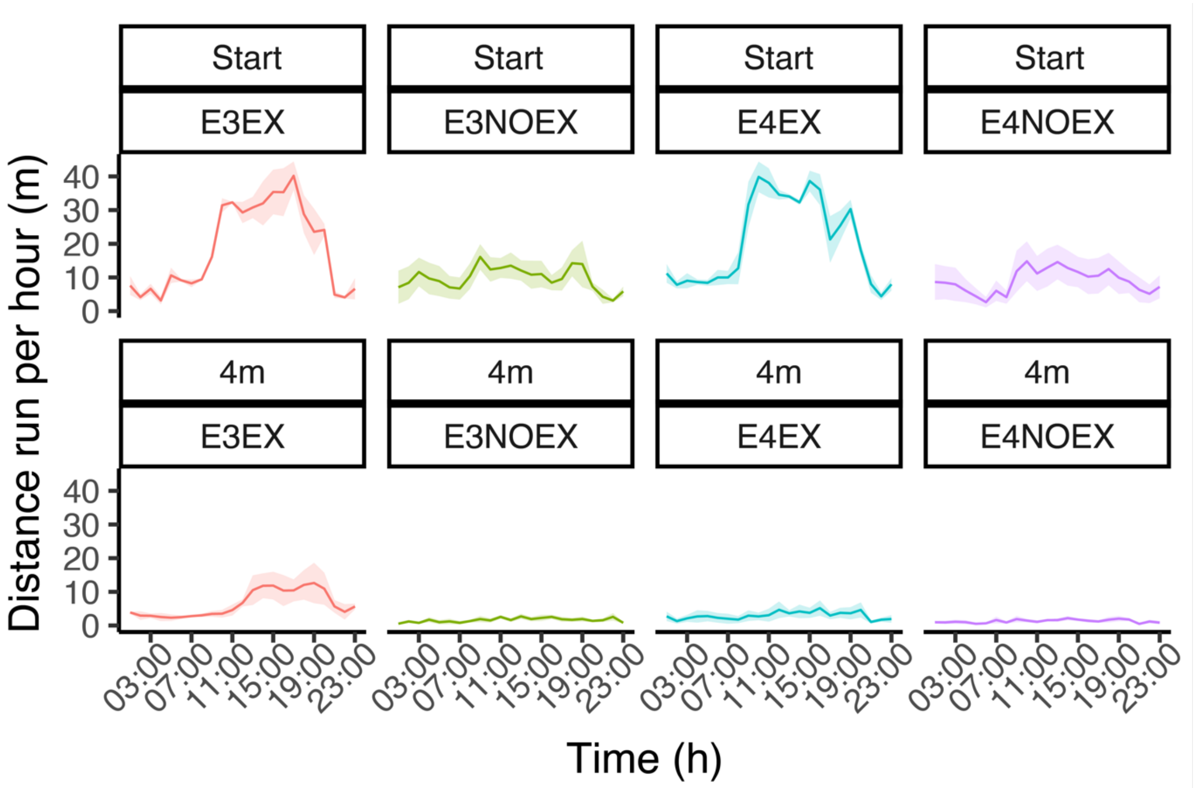

Supplementary Figure 1. Distances travelled per hour of mice in different experimental conditions at the start of the experiment (2 months of age) and after 4 months of differential wheel access. Hourly distances are averaged across 4 days of recordings. Data is mean +/- SEM. (3-6 mice). For details on N and statistical outputs see Supplementary Table 1 in Supplementary Data.

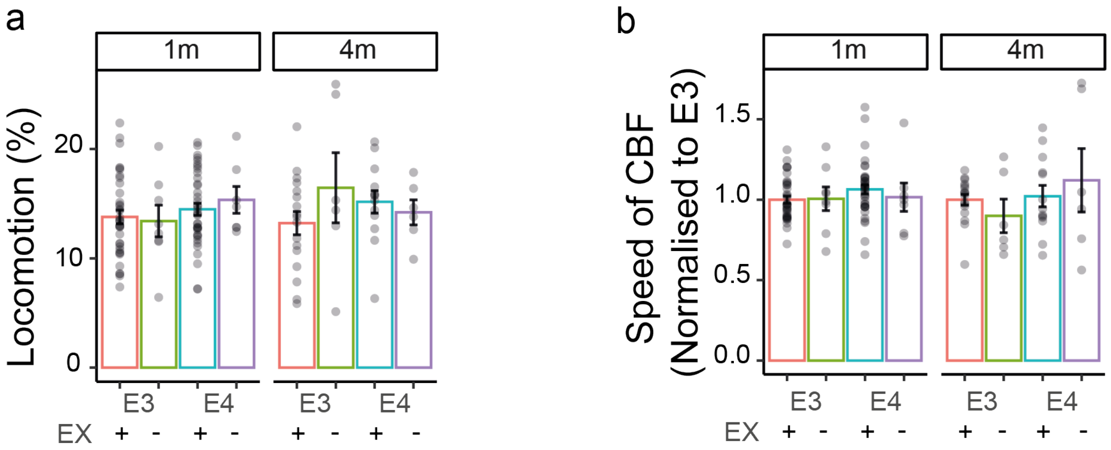

Supplementary Figure 2. There was no difference in locomotion or speed of CBF between groups during resting periods. (a) The percentage of time spent in locomotion during the length of spontaneous net haemodynamic recordings is not different between experimental groups. (b) Speed of CBF at baseline was not affected by genotype, exercise or duration. Dots represent average duration per mouse (N mice: 6-19). Bars indicate mean and error bars indicate mean +/- SEM. For N and statistical outputs see Supplementary Table S1 in Supplementary Data.

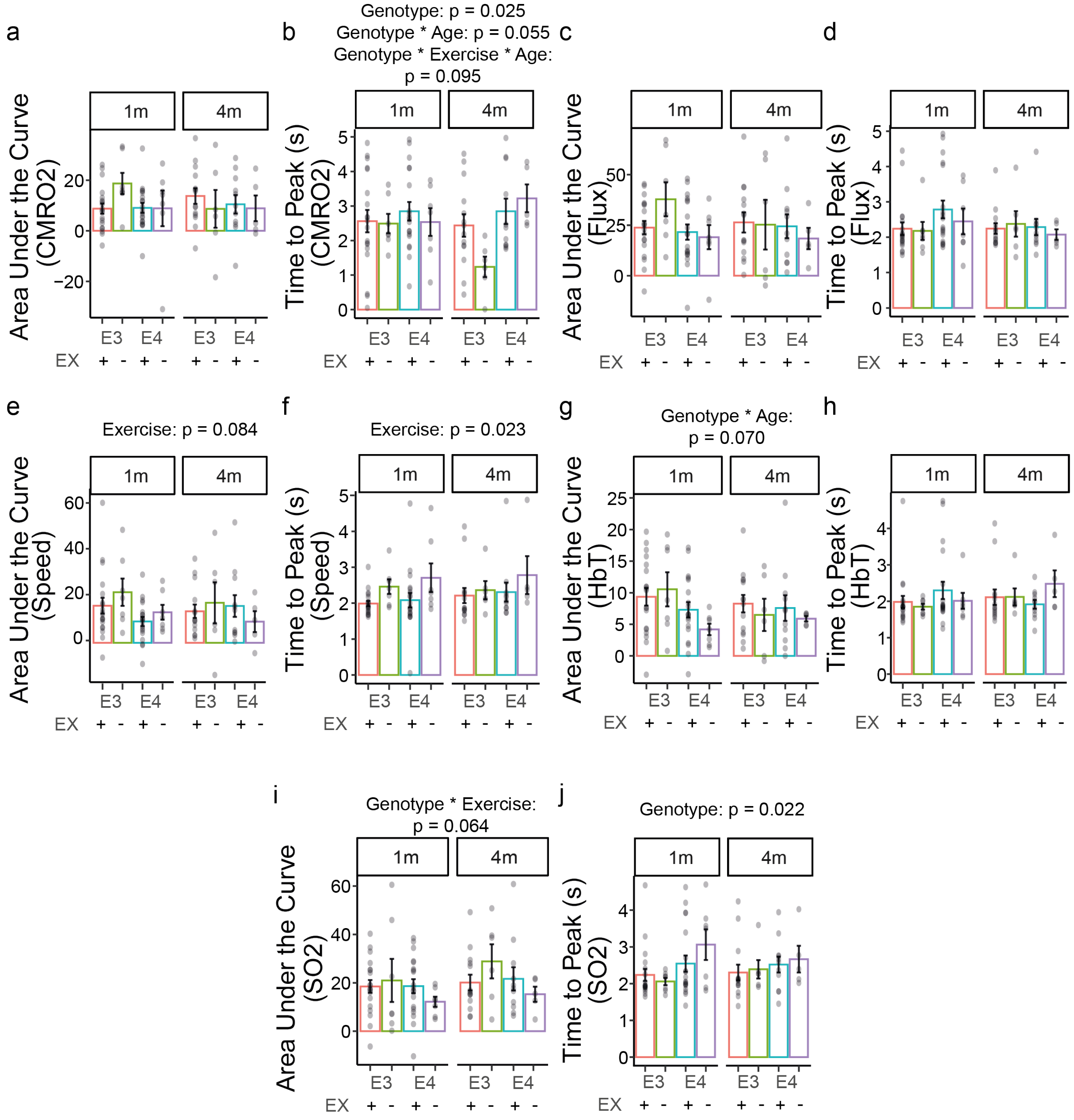

Supplementary Figure 3. Area Under the Curve (AUC) and time to peak of net haemodynamic measures during visual stimulus. (a,c,e,g,i) AUC and (b,d,f,h,j) time to peak response for different haemodynamic measures during visual stimulation. There was no genotype, exercise or duration effect on the AUC of CMRO2 (a) and flux (c), but there was a trend effect of exercise in increasing the Speed of CBF (e), a trend level interaction between genotype and duration on HbT (g) and a trend level interaction between genotype and exercise on sO2 AUC (i). APOE4 mice has lower CMRO2 (b) and sO2 responses (j). Exercise fasted the response time of Speed of CBF (f). Flux (d) and HbT (h) response time was similar between groups. Dots represent average per mouse (N mice: 5-19). Bars and error bars: mean +/- SEM. For N and statistical outputs see Supplementary Table S2 in Supplementary Data.

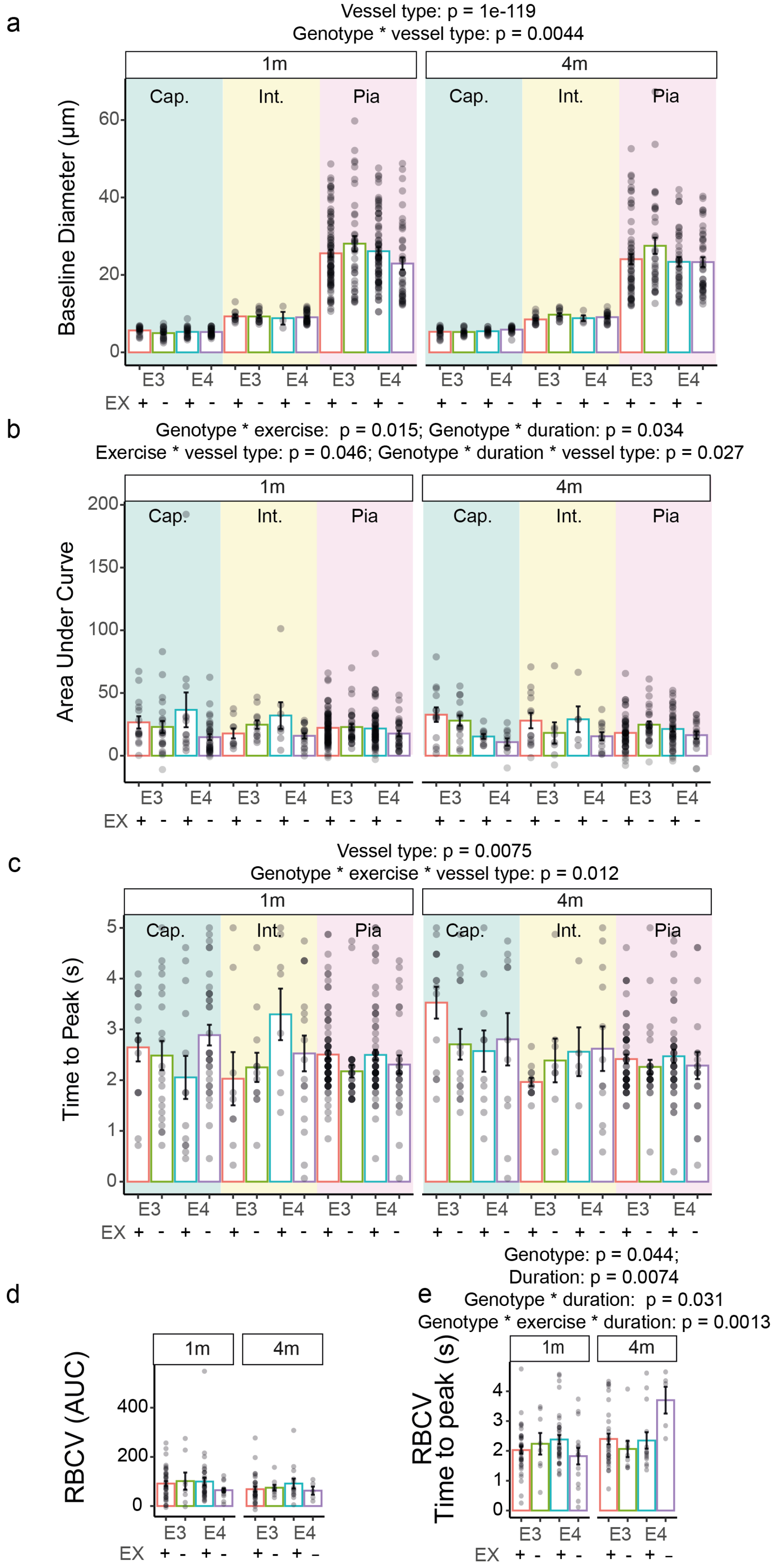
a
Vessel type: p = 1e-119
Genotype * vessel type: p = 0.0044
1m
4m
Cap.
Int.
Pia
Baseline Diameter (µm)
0
20
40
60
E3
E4
EX + - + -
b
Genotype * exercise: p = 0.015; Genotype * duration: p = 0.034
Exercise * vessel type: p = 0.046; Genotype * duration * vessel type: p = 0.027
Area Under Curve
0
50
100
150
200
c
Vessel type: p = 0.0075
Genotype * exercise * vessel type: p = 0.012
Time to Peak (s)
0
1
2
3
4
5
d
RBCV (AUC)
0
200
400
e
Genotype: p = 0.044;
Duration: p = 0.0074
Genotype * duration: p = 0.031
Genotype * exercise * duration: p = 0.0013
RBCV
Time to peak (s)

Supplementary Figure 4. Additional properties of different vascular segments. (a) Vessel diameter at baseline in pial arteries (34-93 vessels from 5-19 mice), intermediate vessels (3-33 vessels from 3-7 mice) and capillaries (16-30 capillaries from 3-6 mice). Vessels sampled were the same size across genotype, exercise or exercise duration groups, except for APOE3 pial vessels that were slightly larger. (b) Area Under the Curve (AUC) of vessel responses during visual stimulation for pial, intermediate vessels and capillaries. (c) Time taken for pial, intermediate vessels and capillaries to reach their maximum dilation during neuronal activity (time to peak). (d) There was no difference between conditions or exercise time points on RBCV AUC. (e) APOE4 mice had slower increases in RBCV, which were worsened by age and no exercise. (29-88 pial vessels from 5-19 mice; 5-19 intermediate vessels from 3-6 mice; 10-33 capillaries from 3-7 mice; 11-47 RBCVs from 3-15 mice). Bars and errors bars: mean +/-SEM. For N and statistical outputs see Supplementary Table S3 in Supplementary Data.

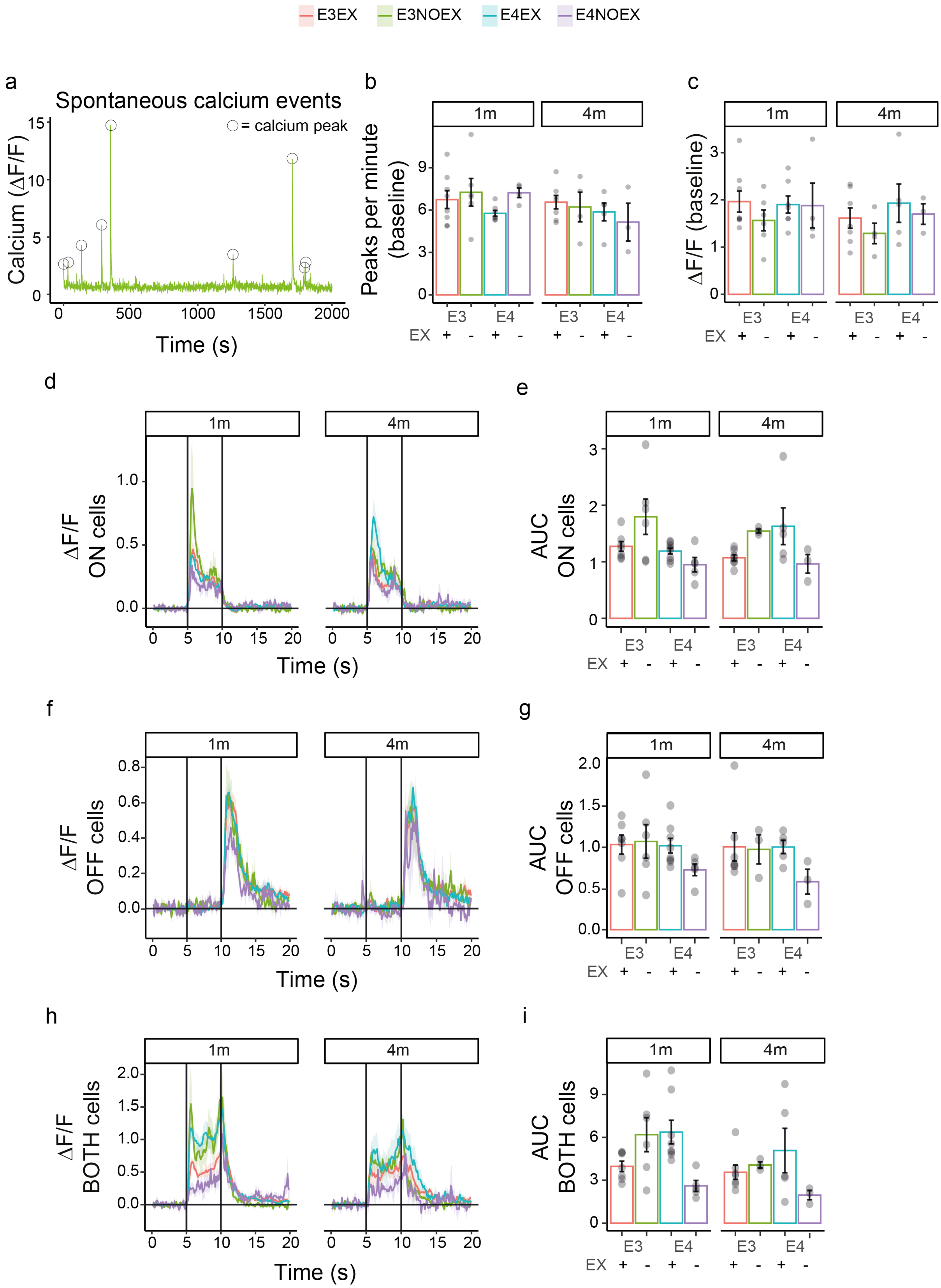

Supplementary Figure 5. Baseline neuronal activity is similar between experimental groups but APOE4 sedentary mice have lower activation of neurons that respond during stimulus onset and offset. (a) Example trace of spontaneous calcium events during baseline recordings. Peaks (indicated by circles) were identified as

changes in signal larger than at least 2 s.d. from average baseline signal. (b) The number of detected peaks per minute during baseline did not differ between genotypes, exercise conditions or duration nor did the size of the spontaneous neuronal activity events (c) (N mice: 3-8). Classifying neurons based on their responses during stimulus as ON (d,e) (when they activate as the stimulus comes on), OFF (f,g) (when they activate as the stimulus comes off) and BOTH (h,i) (when they activate as the stimulus comes on and as the stimulus comes off) we saw that APOE4 sedentary mice had BOTH neurons that activated more weakly compared to the other groups (i). Dots representing average $\Delta F/F$ per animal. (N mice: 3-8). Bars and error bars: mean +/- SEM. For N and statistical outputs see Supp Table S4 in Supplementary Data.

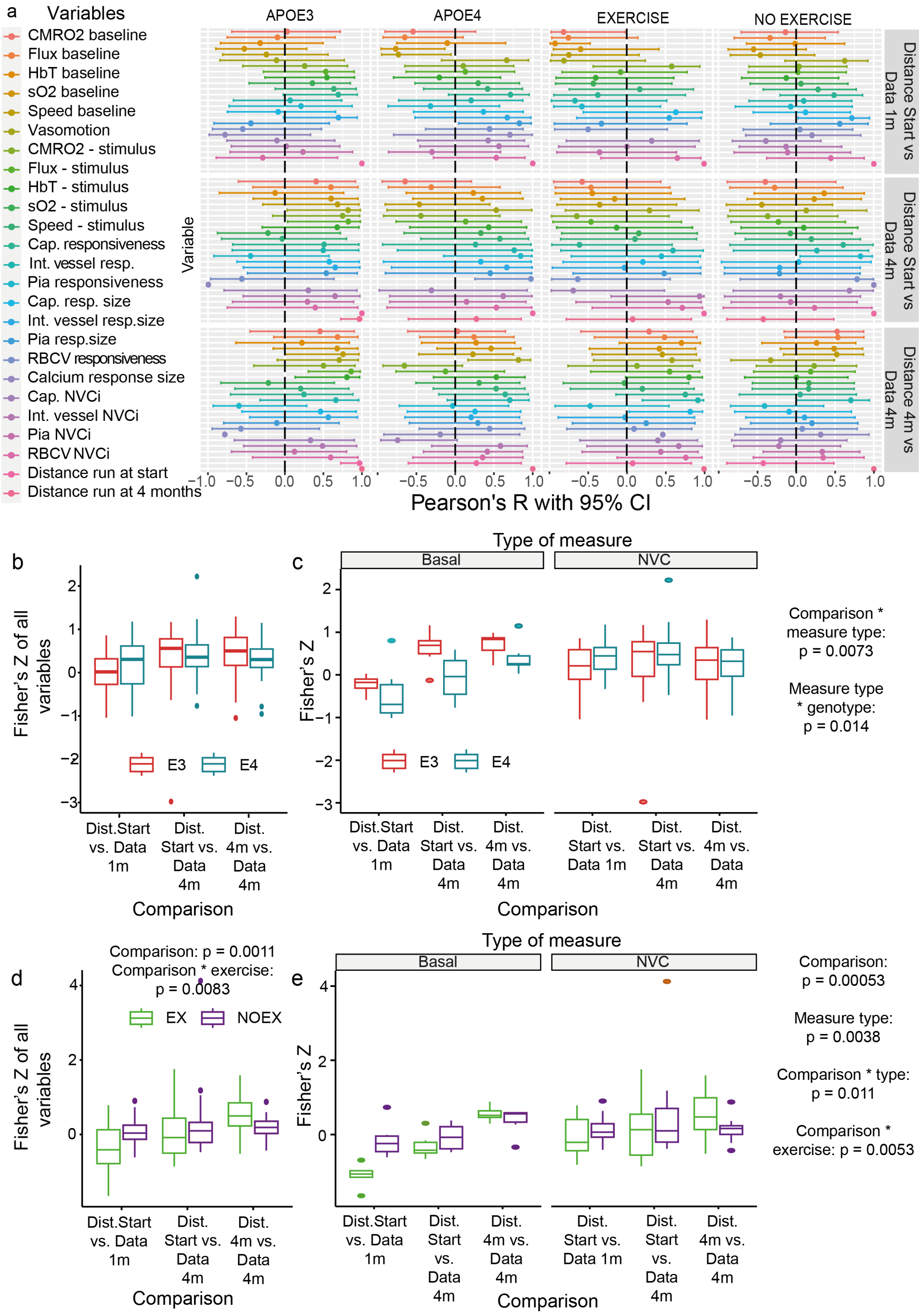

Supplementary Figure 6. Correlations between neurovascular function and exercise plotted by genotype and exercise groups. (a) Data shows Pearson's R with 95% confidence intervals (CI) calculated per genotype or

exercise group (n = 2-10 mice) for the different variables measured at 3- and 6-month of age correlated with the distance run by the mice at 2- and 6-month*s* of age. (b) Box plots of the distribution of correlation coefficients calculated for each genotype (b,c) or exercise group (d,e) transformed into Fisher's Z values, across all variables (b,d) or split into measures of basal neurovascular function and NVC (c,e). P values show significant results of ANOVA (b,d:condition * measure type,c: condition * measure * genotype; e: condition * measure * exercise group) on Fisher's Z. Full statistical results are in Supplementary Table S5 in Supplementary Data. Genotype affects correlations of exercise with basal but not NVC measures (measure type * genotype is significant), while correlations with degree of exercise do not improve over time in the no exercise group (comparison * exercise is significant).

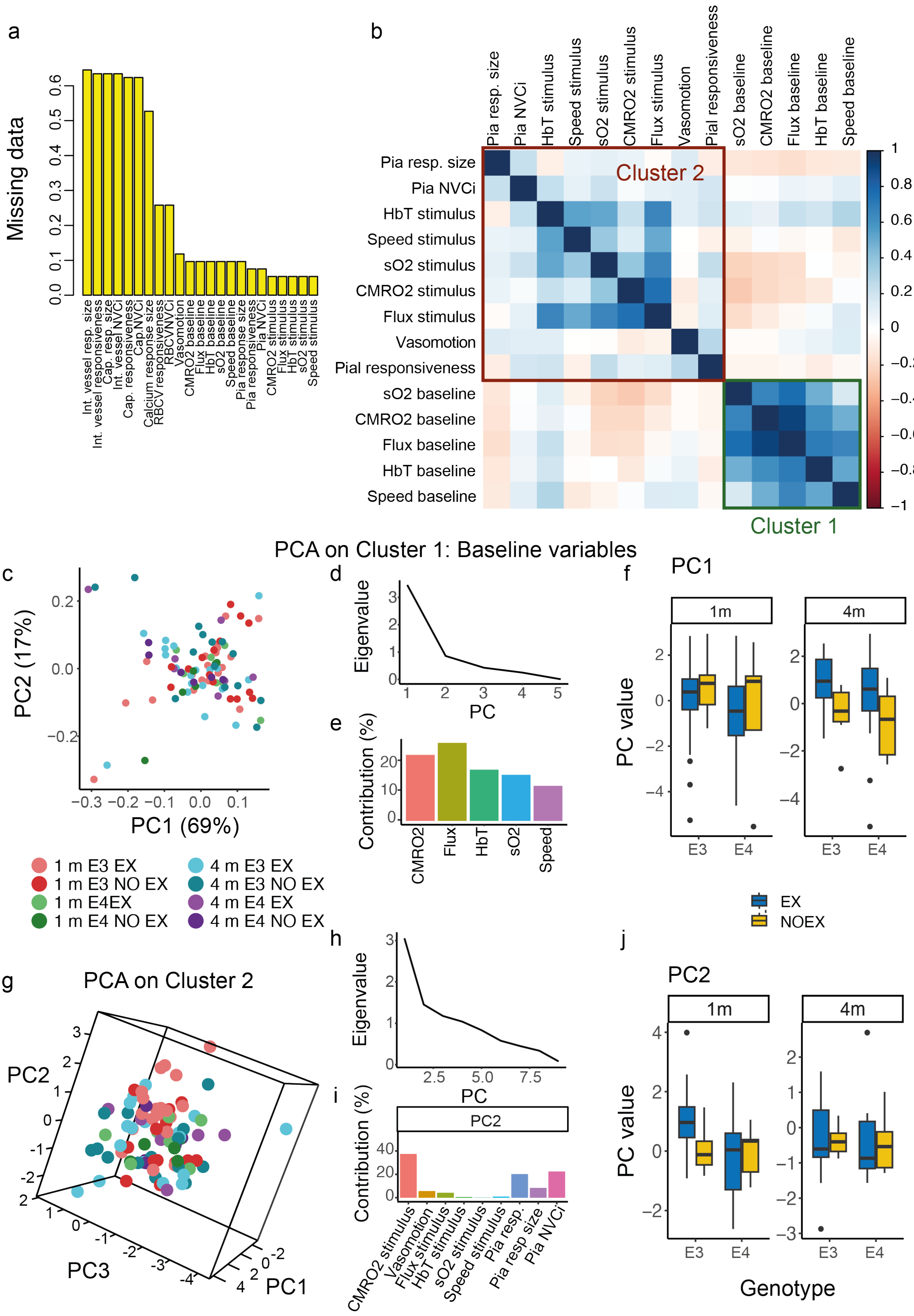
a
Missing data
0.0
0.1
0.2
0.3
0.4
0.5
0.6
Int. vessel resp. size
Int. vessel responsiveness
Cap. resp. size
Int. vessel NVCi
Cap. responsiveness
Cap.NVCi
Calcium response size
RBCV responsiveness
RBCVNVCi
Vasomotion
CMRO2 baseline
Flux baseline
HbT baseline
sO2 baseline
Speed baseline
Pia responsiveness
Pia response size
Pia NVCi
CMRO2 stimulus
Flux stimulus
HbT stimulus
sO2 stimulus
Speed stimulus
b
Pia resp. size
Pia NVCi
HbT stimulus
Speed stimulus
sO2 stimulus
CMRO2 stimulus
Flux stimulus
Vasomotion
Pial responsiveness
sO2 baseline
CMRO2 baseline
Flux baseline
HbT baseline
Speed baseline
Cluster 2
Cluster 1
1
0.8
0.6
0.4
0.2
0
−0.2
−0.4
−0.6
−0.8
−1
PCA on Cluster 1: Baseline variables
c
PC2 (17%)
PC1 (69%)
0.2
0.0
−0.2
−0.3 −0.2 −0.1 0.0 0.1
1 m E3 EX
1 m E3 NO EX
1 m E4EX
1 m E4 NO EX
4 m E3 EX
4 m E3 NO EX
4 m E4 EX
4 m E4 NO EX
d
Eigenvalue
PC
1 2 3 4 5
e
Contribution (%)
CMRO2
Flux
HbT
sO2
Speed
f
PC1
1m
4m
PC value
E3
E4
EX
NOEX
g
PCA on Cluster 2
PC2
PC3
PC1
h
Eigenvalue
PC
2.5 5.0 7.5
i
Contribution (%)
PC2
CMRO2 stimulus
Vasomotion
Flux stimulus
HbT stimulus
sO2 stimulus
Speed stimulus
Pia resp.
Pia resp size
Pia NVCi
j
PC2
1m
4m
PC value
E3
E4
Genotype

Supplementary Figure 7. Principal Component Analysis (PCA) over to identify underlying factors driving inter-variable correlations do not show any effect of exercise and genotype. (a) Percentage of missing data for each animal. Animals that had >20% data missing were excluded from PCA analysis. (b) Correlation matrix of the neurovascular values studies showed the presence of two clusters of correlated variables, with one comprising the baseline haemodynamic measurements and the other including all other variables. (c) Plot of the first two principal components (PC) identified by conducting a PCA over the first cluster. PC1 accounts for 69% of the total variance and PC2 accounts to 17%. There was no clear distinction between the experimental groups. (d) Only PC1 had an eigenvalue over 1. (e) PC1 had similar contributions from all baseline variables. (f) PC1 value was not affected by genotype, exercise or duration. (g) Distribution of PC1-3 obtained from conducting PCA on the second cluster. (h) PC1-4 had an eigenvalue higher than 1. Correction for multiple comparison using Bonferroni correction revealed that the only significant effect was found in PC2. (i) Vasomotion, pial responsivity and pial NVCi were the main contributing values for PC2. (j) PC2 was significantly affected by duration, with APOE genotype trending towards significance. See Supplementary Table S6 in Supplementary Data for statistical detail
