# Supplementary material for: APOE4 and sedentary lifestyle synergistically impair neurovascular function in the visual cortex of awake mice
Source: Commun Biol. 2025 Jan 29;8:144. doi: 10.1038/s42003-025-07585-z (PMC11779976; doi:10.1038/s42003-025-07585-z)
Supplement: Supplementary file 3 — Description of Additional Supplementary Files [file 42003_2025_7585_MOESM3_ESM.pdf]

# Description of Additional Supplementary Files

Filename: Supplementary Data

Description: Full statistical reports for all analyses presented in all figures and/or reported elsewhere in the manuscript.
