# Supplementary material for: APOE4 and sedentary lifestyle synergistically impair neurovascular function in the visual cortex of awake mice
Source: Commun Biol. 2025 Jan 29;8:144. doi: 10.1038/s42003-025-07585-z (PMC11779976; doi:10.1038/s42003-025-07585-z)
Supplement: Supplementary file 4 — Reporting Summary [file 42003_2025_7585_MOESM4_ESM.pdf]

Corresponding author(s): DAPR Manuscript COMMSBIO-24-3325-T

Last updated by author(s): Jun 17, 2024

Reporting Summary

Nature Portfolio wishes to improve the reproducibility of the work that we publish. This form provides structure for consistency and transparency in reporting. For further information on Nature Portfolio policies, see our Editorial Policies and the Editorial Policy Checklist.

Statistics

For all statistical analyses, confirm that the following items are present in the figure legend, table legend, main text, or Methods section.

n/a	Confirmed	
☐	☒	The exact sample size (*n*) for each experimental group/condition, given as a discrete number and unit of measurement
☐	☒	A statement on whether measurements were taken from distinct samples or whether the same sample was measured repeatedly
☐	☒	The statistical test(s) used AND whether they are one- or two-sided<br>*Only common tests should be described solely by name; describe more complex techniques in the Methods section.*
☐	☒	A description of all covariates tested
☐	☒	A description of any assumptions or corrections, such as tests of normality and adjustment for multiple comparisons
☐	☒	A full description of the statistical parameters including central tendency (e.g. means) or other basic estimates (e.g. regression coefficient) AND variation (e.g. standard deviation) or associated estimates of uncertainty (e.g. confidence intervals)
☐	☒	For null hypothesis testing, the test statistic (e.g. *F*, *t*, *r*) with confidence intervals, effect sizes, degrees of freedom and *P* value noted<br>*Give P values as exact values whenever suitable.*
☒	☐	For Bayesian analysis, information on the choice of priors and Markov chain Monte Carlo settings
☐	☒	For hierarchical and complex designs, identification of the appropriate level for tests and full reporting of outcomes
☐	☒	Estimates of effect sizes (e.g. Cohen's *d*, Pearson's *r*), indicating how they were calculated

Our web collection on statistics for biologists contains articles on many of the points above.

Software and code

Policy information about availability of computer code

Data collection	A custom code written using Bonsai.rx was used to control the cameras, acquiring 4-hour long videos (240x360 pixels) for 72 hours consecutively at a rate of 30 frames per second.<br>Ex-vivo brain slices were imaged using a Leica SP8 TCS confocal microscope (Leica Microsystems) using a 20x air objective (HC PL APO CS2 20X/0.75, Leica Microsystems). Continuous wave lasers with excitation wavelength of 488nm and 555nm were used to collect 1024x1024 pixel matrix Z-stacks of FITC-labelled brain vasculature and NG2-DsRed pericytes from V1 and CA1, with a line average of 4 and pixel size of 0.45x0.45x1 µm.<br>A combined laser Doppler Flowmetry/haemoglobin spectroscopy probe (Oxy-CBF probe; VMS OXY/LDF Moor instruments) was used to record net haemodynamic measures across a tissue volume of around 500x500x1000µm 58 at a frequency of 40 Hz from the visual cortex of the mice.<br>SciScan software (Scientifica) was used to collect two-photon recordings of vascular function.
Data analysis	Code used to extract data from images is available at https://github.com/BrainEnergyLab. Code used to generate each figure from these data is available at https://figshare.com/s/df4a31aa60e275bd73b6. These repositories will be archived and a doi to the specific version created prior to publication of this manuscript.

For manuscripts utilizing custom algorithms or software that are central to the research but not yet described in published literature, software must be made available to editors and reviewers. We strongly encourage code deposition in a community repository (e.g. GitHub). See the Nature Portfolio guidelines for submitting code & software for further information.

Data

Policy information about availability of data

All manuscripts must include a data availability statement. This statement should provide the following information, where applicable:

- Accession codes, unique identifiers, or web links for publicly available datasets
- A description of any restrictions on data availability
- For clinical datasets or third party data, please ensure that the statement adheres to our policy

Processed data used to create each figure is available at https://figshare.com/s/df4a31aa60e275bd73b6. Raw images are available from the authors on request.

Research involving human participants, their data, or biological material

Policy information about studies with human participants or human data. See also policy information about sex, gender (identity/presentation), and sexual orientation and race, ethnicity and racism.

Reporting on sex and gender	*Use the terms sex (biological attribute) and gender (shaped by social and cultural circumstances) carefully in order to avoid confusing both terms. Indicate if findings apply to only one sex or gender; describe whether sex and gender were considered in study design; whether sex and/or gender was determined based on self-reporting or assigned and methods used. Provide in the source data disaggregated sex and gender data, where this information has been collected, and if consent has been obtained for sharing of individual-level data; provide overall numbers in this Reporting Summary. Please state if this information has not been collected. Report sex- and gender-based analyses where performed, justify reasons for lack of sex- and gender-based analysis.*
Reporting on race, ethnicity, or other socially relevant groupings	*Please specify the socially constructed or socially relevant categorization variable(s) used in your manuscript and explain why they were used. Please note that such variables should not be used as proxies for other socially constructed/relevant variables (for example, race or ethnicity should not be used as a proxy for socioeconomic status). Provide clear definitions of the relevant terms used, how they were provided (by the participants/respondents, the researchers, or third parties), and the method(s) used to classify people into the different categories (e.g. self-report, census or administrative data, social media data, etc.) Please provide details about how you controlled for confounding variables in your analyses.*
Population characteristics	*Describe the covariate-relevant population characteristics of the human research participants (e.g. age, genotypic information, past and current diagnosis and treatment categories). If you filled out the behavioural & social sciences study design questions and have nothing to add here, write "See above."*
Recruitment	*Describe how participants were recruited. Outline any potential self-selection bias or other biases that may be present and how these are likely to impact results.*
Ethics oversight	*Identify the organization(s) that approved the study protocol.*

Note that full information on the approval of the study protocol must also be provided in the manuscript.

Field-specific reporting

Please select the one below that is the best fit for your research. If you are not sure, read the appropriate sections before making your selection.

☒ Life sciences ☐ Behavioural & social sciences ☐ Ecological, evolutionary & environmental sciences

For a reference copy of the document with all sections, see nature.com/documents/nr-reporting-summary-flat.pdf

Life sciences study design

All studies must disclose on these points even when the disclosure is negative.

Sample size	Sample size was decided based on the expected size of the effects observed.
Data exclusions	No data was excluded except for the extracted exercise data, where the distances travelled within 10-minutes that were at the top and bottom 1% of the distribution were filtered out to exclude extreme outliers (which occurred when there was a failure to classify a frame correctly).
Replication	The in vivo vascular data collected from mice in cohort two mostly replicates the data collected and published in Bonnar, Shaw et al. 2023 (cohort 3). The same methods were used and mice of similar age were involved in the study. To ensure replicability, we described thoroughly the methods involved in the study and made available all analysis codes used.
Randomization	APOE3-TR and APOE4-TR mice were randomly given an exercise wheel or were housed without. Initial randomisation was achieved by blindly selecting a wheel or no wheel condition for the first mouse in a litter of a given sex to undergo surgery. The next mouse from that litter and sex was then provided with the opposite condition to match littermates across conditions. If there were more mice of a given litter and sex used for the experiment then the experimental condition was again randomly selected for the next mouse, which was then counterbalanced

	by the following mouse, and so on. Later in the experiment, if there were fewer mice in a given experimental group, then mice were prioritised for the conditions where the sample size was smaller.
Blinding	For vascular density analysis and exercise analysis, experimenters were blinded to the genotype and exercise condition of the animals. For in vivo recordings using two-photon microscopy and Oxy-CBF probe, the experimenter was not blind to genotype or exercise group.

Reporting for specific materials, systems and methods

We require information from authors about some types of materials, experimental systems and methods used in many studies. Here, indicate whether each material, system or method listed is relevant to your study. If you are not sure if a list item applies to your research, read the appropriate section before selecting a response.

Materials & experimental systems

n/a	Involved in the study
☒	☐ Antibodies
☒	☐ Eukaryotic cell lines
☒	☐ Palaeontology and archaeology
☐	☒ Animals and other organisms
☒	☐ Clinical data
☒	☐ Dual use research of concern
☒	☐ Plants

Methods

n/a	Involved in the study
☒	☐ ChIP-seq
☒	☐ Flow cytometry
☒	☐ MRI-based neuroimaging

Animals and other research organisms

Policy information about studies involving animals; ARRIVE guidelines recommended for reporting animal research, and Sex and Gender in Research

Laboratory animals	C57BL/6J-Tg(Thy1-GCaMP6f)GP5.5Dkim/J mice were crossed with APOE-TR (B6.129P2-Apoetm3(APOE*4)Mae N8 or B6.129P2-Apoetm2(APOE*3)Mae N8) mice for in vivo recordings of vasculature, whereas NG2-DsRed mice (Tg(Cspg4-DsRed.T1)1Akik/J) were crossed with APOE-TR mice for ex vivo vasculature and pericyte analysis. The mice involved in the study were 9 weeks to 6 months of age.
Wild animals	n/a
Reporting on sex	Mice of both sexes were used and experimental groups had similar numbers of females and males. Sex was considered as a variable. This study used three cohorts of mice. Each cohort contained mice of both sexes. Analysis of sex dependent effect (LMM with sex as fixed factor) found only a few significant sex effects, which did not point toward a clear direction of effects. Summary of observed sex effects (where present) can be found in Supplementary Table 7 and all statistical outputs are in Supplementary Table S7.
Field-collected samples	n/a
Ethics oversight	Procedures involving animal use were conducted in accordance with the UK Animals (Scientific Procedures) Act 1986. Local approval was granted by the University of Sussex Animal Welfare and Ethical Review Board.

Note that full information on the approval of the study protocol must also be provided in the manuscript.

Plants

Seed stocks	n/a
Novel plant genotypes	n/a
Authentication	n/a
