# Supplementary material for: APOE4 and sedentary lifestyle synergistically impair neurovascular function in the visual cortex of awake mice
Source: Commun Biol. 2025 Jan 29;8:144. doi: 10.1038/s42003-025-07585-z (PMC11779976; doi:10.1038/s42003-025-07585-z)
Supplement: Supplementary file 5 — Peer Review file [file 42003_2025_7585_MOESM5_ESM.pdf]

nature portfolio

Peer Review File

APOE4 and sedentary lifestyle synergistically impair neurovascular function in the visual cortex of awake mice

Corresponding Author: Professor Catherine Hall

Version 0:

Reviewer comments:

Reviewer #1

(Remarks to the Author)
"APOE4 and sedentary lifestyle synergistically impair neurovascular function in the visual cortex of awake mice"

By Anderle…Hall.

APOE4 is the strongest genetic risk factor for Alzheimer's disease, and humans and mouse models carrying human APOE4 develop vascular changes compared to APOE3 carriers. Here the authors investigate the effects of voluntary exercise in mice carrying the human APOE4 allele compared to those carrying the human APOE3 allele. Mice were provided with or without a running wheel in their home cages for exercise. At 3 and 6 months of age neuronal and vascular function were measured in vivo in awake mice with two-photon microscopy, as well as hemodynamic measures and tissue oxygenation. Vascular density measures were made ex vivo. The authors found that while the AOPE4 mice tended to have deficits in neurovascular function, exercise ameliorated many of these deficits. The exercise tended to have more modest effects on the APOE3 mice.

This is a technically challenging study that provides important observations of the effects of exercise on APOE4-mediated physiological changes. However, there are several points that should be addressed that would help to improve and clarify the study prior to publication. Specific comments are below.

Major:

1. In lines 142-143, the authors indicated that capillary densities may be linked to sO2 values. It would be informative to plot the correlation and perform correlation analysis to determine if these two parameters are related as the authors suggest. Otherwise, this link remains somewhat hypothetical.

2. In lines 148-149, what is the significance of comparing HC to V1 capillary density? This does not seem to be presented in the accompanying figure (fig. 2b) or main text.

3. The observations that neurovascular metrics are impaired in APOE4 mice (e.g. Figure 5) are consistent with reports of pericyte dysfunction and endothelial changes with age in APOE4 mice, with vascular changes preceding overt neuronal changes. See e.g. PMID: 36040482. Do the authors observe any mural cell or endothelial changes with age in these mice (other than vascular density)? Does exercise have any affect on these changes, if present?

4. Related to the comment above, APOE4 carriers and mice develop blood-brain barrier (BBB) breakdown or increased permeability. Did mice that exercised show any improvements in BBB integrity?

5. The pattern of the responses between genotypes in Figure 4k seems quite different between the 3m and 6m groups. Can the authors comment on what might cause this? Does the effect of APOE4 genotype overwhelm any other effects of exercise as these mice age?

6. Although not explicitly stated, I assume that larger NVCi values are better? Some description of how to interpret this metric devised by the authors would be helpful.

7. Many of the Figure 4 panels are not mentioned in the related results section (lines 210-252). Please update this section to describe the data illustrated, or update the figure to match this section of the text. As presented, it is difficult to follow.

8. In the figures, it is difficult to tell which groups are significantly different from each other. This makes it more difficult to evaluate the data presented. It would be helpful to provide markers or bars to guide the reader to what conditions differ from each other.

9. Although the reader is referred to the supplemental tables for exact n-numbers, it would be informative to at least include an n-number range in the figure captions, particularly because some of the data is presented as n = number of mice, and n = other measure such as tissue slices, which does not inform about number of mice used. This needs to be more transparent in the figure captions.

Minor:

10. Some figure captions seem to include a lot of exposition/interpretation about the results presented which would be more appropriate to include in the main text.

11. Age groups are not labeled in the figure 3 bar graphs.

12. Fisher's Z score does not appear to be described in the methods.

Reviewer #2

(Remarks to the Author)
This novel study by Anderle et al reports on understandings on the impact of the APOE4 genotype with the specific lifestyle contribution of exercise. The study builds on work on understanding the impact of APOE4 by Bonnar et al who reported impaired vascular responsiveness and vasomotion in awake mice. The experiments in this study were all conducted in awake mice, therefore providing an important contribution to the field and in vivo experimental community, free from confounding effects by anaesthesia. The major claim that a lack of exercise in association with the APOE4 genotype is detrimental to cerebral vascular reactivity and function is an important one, on which further research is needed to identify the specific mechanisms, as the authors have identified in the discussion.

The experimental design is impressive, and the range of data collected/techniques used leads to a thorough conclusion important and of interest to the cerebral vascular field.

One general comment is the age range (2/3 and 6 months) used to create age as a variable, as according to https://www.jax.org/research-and-faculty/research-labs/the-harrison-lab/gerontology/life-span-as-a-biomarker# (Figure V.3) 3-6 months still equates to 'mature adult' of 2-30 years, making it tricky to use age as a variable. Perhaps a solution could be for the authors could be more explicit about the age equivalent in human years, or refer to this variable in the text as duration of exercise?

Did the authors record the weight of the mice to check if exercise was causing the mice to be leaner/whether there were any differences in weight between the 2 genotypes?

Please see below for specific comments:

-Line 43: please could the authors add a reference for the statement about the weak binding of APOE4 to LRP-1 as this is not fully described in reference 6 (Montague et al) or use a more specific reference (eg Halliday et al 2016 PMID 25757756)
-Line 71: please could the authors state how they decided on the duration of housing with an exercise wheel for '1-2 or 4-5 months'
-Figure 1a: please could the authors change the age in weeks at time of surgery to months to match the latter three timepoints for clarity
-Please could the authors state why exercise and neurovascular function were recorded separately at 2 and 3 months, but together at 6 months?
-Figure 1 f and g: if possible, please could the authors consider putting in an axis break on the y axis to highlight the difference in distance run between 2 months and 6 months?
-Figure 1 h: according to supplementary table 1, the n number for 2 months E3EX is n=3, the figure legend suggests that each dot represents an animal, but there are more dots on the graph – please could the authors clarify what the dots represent?
-Line 150, figure 2 legend: please could the authors clarify if each brain slice is from one animal
-Lines 139 – 142: please could the authors correct the figures referred too (eg flux on line 139 should refer to Fig 2 c instead of Fig 2 d)
-Please could the authors clarify which region the capillary density was measured in, as in line 176, it states hippocampus and line 127 visual cortex. Please also check line 149 for the description of the region in the figure legend.
-Please could the authors match the x axis on Fig 3c for clarity?
-Line 234: please could the authors check the p value for the main effect of exercise on responsiveness as the values in the supp table state p=0.0236 rather than p=0.017, and also the p value for genotype line 235.
-Line 235-236: please could the authors clarify whether the effect by APOE4 impacted by exercise is across vessel type or just one vessel type (as the p value is not in the supplementary table)

-Lines 247 – 248: please could the authors check the p values as they do not match up to the p values in the figure or supplementary table
-Lines 255-270: The figure legend for Figure 4 is a little unclear; please could the authors check the corresponding letters?
-Figure 5: the results from the NVCi calculations are very interesting – would it be possible for the authors to add p values to the graphs to highlight the effect of exercise and genotype across the vessel types?
-Figure 6: please could the authors clarify whether the comparison of distance run at 2 months with data from mice at 6 months are between the same mice ie are these the same mice being compared throughout the study?
-When referring to supplementary figure 6 between lines 342 – 357, please could the authors add the letter for each part of the figure referred to for clarity?
-Discussion Line 367: the phrase 'different levels of voluntary exercise' could potentially be misleading as it could suggest easy/difficult exercises; could the authors consider reframing eg by saying 'different periods of exercise' or something similar?
-Discussion Line 443-444: the references are in a different format (not numbered). Also, for reference 42 at the end of line 444, as the results suggested that exercise was not beneficial for the capillary bed, please could the authors comment on the implications of that reference (where ROS stimulated ET-1 constricts capillary pericytes) in the context of their findings?
-It is interesting that despite the decrease in exercise activity at 6 months compared to 2 months (Figure 1), there is still preservation of neurovascular coupling responses in the APOE4 mice (Figure 5). Do the authors have any suggestions as to why this may be the case?
-Line 738: the figshare link for processed data is broken.

Reviewer #3

(Remarks to the Author)
This manuscript examines the neurovascular response in APOE 3 and 4 mice with or without exercise. Numerous variables were measured yet clarity on the mechanisms related to APOE4 and vascular responses were not overtly elucidated. The current manuscript adds to the available literature and provides some modest advances. While the results are perhaps not as straightforward, the authors should be commended for the significant data collection and analyses. The supplementary details are also appreciated and extensive.
1) While sedentary and APOE4 status resulting in worse outcomes is not too surprising, the current manuscript attempts to synthesize their findings cogently, but given the numerous variables assessed, it is difficult to digest the findings in total. The authors should consider a summary Table of sorts that summarizes the many findings to simplify for future readers the key aspects of the reported study.
2) As the postmortem data vessel density was acquired from a previously published cohort, this should be directly noted in the results. In the present format this information is only available at the very end of the manuscript. Along similar lines in Fig 2 legend, there is reference to V1 and hippocampal data but it is not clear what region is being shown in Fig 2b.
3) Fig 3 – Reading the results, the statistical results are noted in the text, but unlike Fig 2 with extensive statistical notation in the figure, figure 3 is mostly devoid of these notations. It is noticed also in other figures. Is there a reason for not being consistent across figures? It would be great to include all statistical notations in the figures. It is appreciated that all the details are in the supplement but would be beneficial to include across all figures.
4) In several figures there are image or histological examples of the anatomy (ie Fig 4 b, f, j,n).. For completeness it would be nice to know what group these images came from - ie APOE3 Ex for example. See Fig 6a as well.
5) The extensive correlations reported and shown in Fig 6 clearly indicate a positive trend in many of the features assessed. The supplement also breaks this down by genotype. But what appears to be missing is a breakdown by genotype X exercise groups. Not sure if this was an oversight or if this was purposely not included. I would recommend adding this to the supplement as a minimum.
6) The discussion spends considerable time on several putative mechanisms that might underlie the findings in the manuscript, including NO (line 458). What is missing is that the source of the NO (vascular, vs neuronal etc) is likely a critical component to this discussion. Could the authors revise to clarify these interactions in light of their results.
7) Also there is no discussion as to the vascular density changes noted in other studies including VEGF mediated pathways and the relative lack of vessel density noted in the current models utilized herein. Also if there are vascular density changes in human APOE4 but not in the models utilized in this study, how do the authors reconcile relevance to human disease.

Minor items:
1) In several locations within the manuscript, some references did not receive the numerical notification – ie line 169
2) Also in the references, there are some references missing page numbers, others have doi's etc. These should be checked carefully.
3) Some aspects of the study a greatly underpowered to study sex effects… and statistical testing is not warranted…. This should be made explicitly clear in the results as well as in the methods.

Version 1:

Reviewer comments:

Reviewer #1

(Remarks to the Author)
The authors have performed additional experiments and analyses to address my comments, as well as providing updates to

the text and discussion. The manuscript is much improved with these additions.

I also appreciate the addition of the statistical information and n-numbers to the figures. This has aided greatly in clarifying the data.

My only comment is for the authors to please check the figure call outs in the text, as I think there are a couple places where there are typos regarding referencing incorrect figures.

No further comments.

Reviewer #2

(Remarks to the Author)
Many thanks to the authors who have responded to all comments. I have a few very minor comments, but feel that the article is ready for publishing:

1. The findings are now discussed with a further emphasis on duration of exercise, renaming the ages at which exercise was assessed as timepoints 1 and 2, which is very helpful in directing the reader to focus on the duration of exercise, rather than the age the mouse has reached whilst completing that duration.
2. The authors have included the weights of the mice which all similarly increased over time across all genotypes, ruling it out as a variable.
3. The authors have added a novel section with data examining the effect of genotype and exercise on pericyte density and diameter. Pericyte density was not significantly altered, and the authors do not explicitly say this but refer to a trend and possibility in significance of the data.
a. In Figure 2d the authors provide a representative image of a capillary with a pericyte -please could the authors provide small images of the FITC gelatin labelled capillary only and the DsRed labelled pericyte next to the merged image so that the reader can get an idea of distance measured from the soma?
b. For the legend of figure 2, the authors have stated that for capillary and pericyte densities in (b, c) the dots represent slices from up to 4 mice, and details can be found in supplementary data table 2. Please could the authors clarify whether statistical analysis was done on the mean brain slices or the n of mice?
4. The additional analyses on the basal and stimulated NVC are really interesting, and the authors have articulated this well in their manuscript, and figure 7 and supp figure 6 are very useful. The inclusion of potential influence of environmental enrichment in the discussion is also helpful.
5. Thank you to the authors for amending the graphs - the additional of p values above panels makes it easier to read the significance of the figures
6. I agree with the authors that the BBB permeability measures are not necessary to include in the paper given the of the data generated given the experimental limitations
7. The authors have added a graphical abstract of the article in Figure 8 which is a helpful and useful summary.

Open Access This Peer Review File is licensed under a Creative Commons Attribution 4.0 International License, which permits use, sharing, adaptation, distribution and reproduction in any medium or format, as long as you give appropriate

credit to the original author(s) and the source, provide a link to the Creative Commons license, and indicate if changes were made.
In cases where reviewers are anonymous, credit should be given to 'Anonymous Referee' and the source.
The images or other third party material in this Peer Review File are included in the article’s Creative Commons license, unless indicated otherwise in a credit line to the material. If material is not included in the article’s Creative Commons license and your intended use is not permitted by statutory regulation or exceeds the permitted use, you will need to obtain permission directly from the copyright holder.
To view a copy of this license, visit https://creativecommons.org/licenses/by/4.0/

Response to reviewers

We would like to thank the reviewers who were very thoughtful and helpful. Their comments have really helped not only to improve the manuscript clarity and interpretation, but also to add new insights via the new analyses that we conducted in response to their suggestions. We have addressed these suggestions as described in detail below, but the main amendments we have made in response to their comments are:

1. We have conducted new analyses to interrogate the role of pericytes in microvascular changes after exercise. These show that exercise but not genotype modify pericyte and microvascular constriction.
2. We have altered the design of several figures to allow statistical results to be displayed above the data.
3. We have renamed the "age" factor as "duration" throughout to highlight that this is not an ageing study but instead studies the effect of length of exposure to differential levels of physical activity.
4. We have conducted new analyses that reveal interesting differences in how exercise level correlates with basal neurovascular function vs. neurovascular coupling. These analyses reveal that neurovascular coupling is correlated with exercise level from the earliest time, while basal measures become more correlated with exercise level over time. Furthermore, APOE4 mice show lower correlations between basal function and exercise level, revealing a new subtle deficit in these mice: they are less able to increase basal neurovascular function by increasing activity, while neurovascular coupling measures can be improved this way.

We respond point by point to the reviewers below.

Reviewer #1 (Remarks to the Author):

APOE4 is the strongest genetic risk factor for Alzheimer's disease, and humans and mouse models carrying human APOE4 develop vascular changes compared to APOE3 carriers. Here the authors investigate the effects of voluntary exercise in mice carrying the human APOE4 allele compared to those carrying the human APOE3 allele. Mice were provided with or without a running wheel in their home cages for exercise. At 3 and 6 months of age neuronal and vascular function were measured in vivo in awake mice with two-photon microscopy, as well as hemodynamic measures and tissue oxygenation. Vascular density measures were made ex vivo. The authors found that while the APOE4 mice tended to have deficits in

neurovascular function, exercise ameliorated many of these deficits. The exercise tended to have more modest effects on the APOE3 mice.

This is a technically challenging study that provides important observations of the effects of exercise on APOE4-mediated physiological changes. However, there are several points that should be addressed that would help to improve and clarify the study prior to publication. Specific comments are below.

Major:

1. In lines 142-143, the authors indicated that capillary densities may be linked to sO2 values. It would be informative to plot the correlation and perform correlation analysis to determine if these two parameters are related as the authors suggest. Otherwise, this link remains somewhat hypothetical.

We thank the reviewer for raising this point. We agree that to prove that sO2 values are linked to capillary density it is necessary to determine if these values are correlated. Unfortunately, our existing data does not allow for this analysis. We did collect z-stacks of vasculature in vivo, to mark the location of vessel recordings to allow the same vessel to be found again, but they had too small a pixel size and a low signal to noise to allow us to trace the vascular network accurately. In light of this, we have added this statement in the main document in lines 167-169.

"Measuring haemodynamic function and capillary density in the same mice would directly test this conclusion, but this was not possible in this study."

2. In lines 148-149, what is the significance of comparing HC to V1 capillary density? This does not seem to be presented in the accompanying figure (fig. 2b) or main text.

We apologise for this oversight which remained from an earlier draft of the manuscript where we included postmortem capillary density data from the hippocampus. We removed this as the rest of the paper concentrates on the visual cortex, so the hippocampus data was not relevant to the rest of the story but had missed this reference to the old data. We have now removed it.

3. The observations that neurovascular metrics are impaired in APOE4 mice (e.g. Figure 5) are consistent with reports of pericyte dysfunction and endothelial changes with age in APOE4 mice, with vascular changes preceding overt neuronal changes. See e.g. PMID: 36040482. Do the authors observe any mural cell or endothelial changes with age in these mice (other than vascular density)? Does exercise have any affect on these changes, if present?

We appreciate the reviewer bringing up this intriguing point. Previously, we had not examined the effects of genotype and exercise on pericyte numbers and function; however, our ex vivo vascular stacks were obtained from mice with fluorescently labeled pericytes (NG2-DsRed mice). We have now conducted a new analysis of pericyte density across different genotype and exercise groups, and have assessed differences in vessel diameter at the location of pericyte somas compared to regions away from them. Interestingly, our analysis revealed a trend-level increase in pericyte density in the cortex of mice that exercised for 1 to 1.5 months, independent of genotype. Furthermore, we observed a trend towards increased baseline capillary diameters in exercised mice, which was significantly larger at the location of pericyte somas compared to sedentary mice. These findings suggest that active mice have more dilated capillaries, potentially due to reduced pericyte contraction, which may be linked to lower levels of ROS and ET-1, and also potentially increased numbers of pericytes. Given the significance of this data, we have now incorporated it into Figure 2 of the manuscript, and data on vasomotion has been relocated to a new Figure 3. The implications of these data are also now considered in the Discussion.

4. Related to the comment above, APOE4 carriers and mice develop blood-brain barrier (BBB) breakdown or increased permeability. Did mice that exercised show any improvements in BBB integrity?

Our experiments were not designed to interrogate BBB permeability, so it is not possible to answer this question conclusively, but we have performed some new analyses on our data to see if any alterations in BBB were apparent. To do this, for each recording session we took the first 10 frames of each recording (over time the parenchymal signal bleaches so we excluded later frames in the recording) and measured the dye intensity inside and outside the vessel. We then calculated the ratio of the intensity outside vs. inside the vessel as an indication of the degree of leakage of dye from the vessel (i.e. the degree of BBB permeability). However, these measurements are confounded by the time since the dye was introduced to the mouse, so that later recordings would be expected to show greater parenchymal leakage than earlier recordings for the same amount of BBB permeability. Because we were not planning to measure BBB function, we had not recorded the time of dye injection. Instead, we calculated the rate of change of the parenchymal vs intravascular

dye intensity ratio over time over a given recording session. The results of this analysis are shown below. There are no significant differences in the rate of change in the intensity of the dye outside the vessel (relative to the dye intensity inside the vessel) across any groups, so no conclusive evidence for any alterations in BBB permeability due to APOE genotype or exercise condition. However, the rates for the APOE4 mice are (non-significantly) higher than those for APOE3 mice (p = 0.13). Thus, it may be that a low-level increase in BBB permeability is present in our APOE4 mice, that may have been detectable if we had designed our experiments to interrogate this. There is no suggestion in these data, however, that exercise impacts extravascular dye accumulation.

Given the lack of conclusive data around BBB permeability changes in these analyses, and the fact that our experiments were not designed to interrogate these changes, we would prefer to leave this analysis out of the paper.

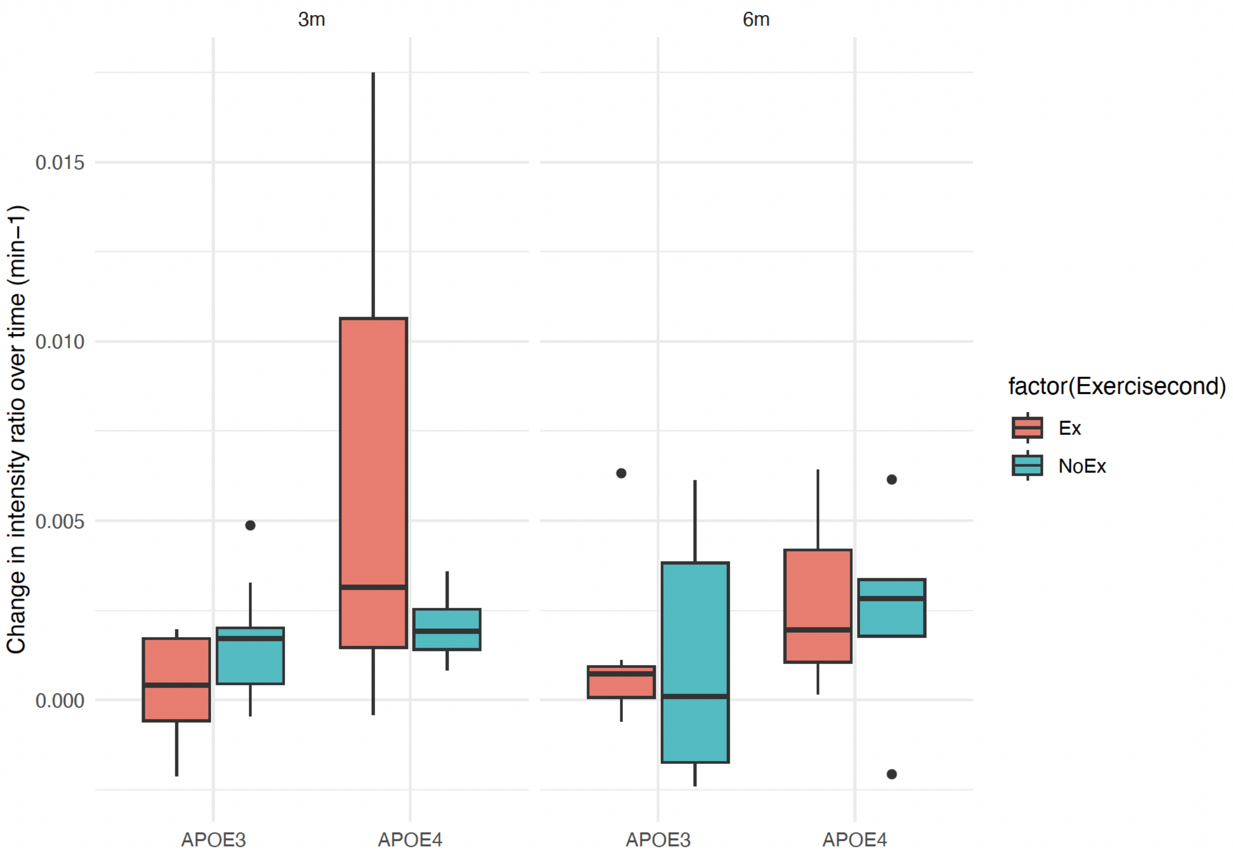

5. The pattern of the responses between genotypes in Figure 4k seems quite different between the 3m and 6m groups. Can the authors comment on what might cause this? Does the effect of APOE4 genotype overwhelm any other effects of exercise as these mice age?

The reviewer raises an interesting point. Our reading of these data is that the capillaries are more sensitive to the negative impact of APOE4 than the pial vessels. This means that, while in pial vessels, increasing exposure to exercise reverses the negative impact of APOE4 genotype, this does not happen in capillaries. This difference across vascular segments suggests that the mechanism of action of exercise is relatively selective for different vascular components and, in the discussion, we speculate that this could be due to the exercise effect being mediated via the NO pathway and therefore not targeting the capillaries to the same degree as arterioles.

6. Although not explicitly stated, I assume that larger NVCi values are better? Some description of how to interpret this metric devised by the authors would be helpful.

We apologise for the confusion. We see that we have not explained the NVCi sufficiently. The reviewer is correct that a larger NVCi indicates stronger neurovascular coupling (i.e. a larger vascular response for a certain amount of neuronal activity). We have added the following sentence to explain this in the results text about Figure 5 (now Figure 6) at lines 352-354:

"Thus, a reduction in NVCi indicates that there is a smaller vascular response for the same amount of neuronal activity, and that there is a deficit in neurovascular coupling."

7. Many of the Figure 4 panels are not mentioned in the related results section (lines 210-252). Please update this section to describe the data illustrated, or update the figure to match this section of the text. As presented, it is difficult to follow.

We apologise that this was unclear and agree that more direct referencing of the figure panels is required. We have now amended the paragraph describing Figure 4 (now Figure 5 and lines 259-298) to insert references to all figure panels, rewording in places to make the link to the figures clearer.

8. In the figures, it is difficult to tell which groups are significantly different from each other. This makes it more difficult to evaluate the data presented. It would be helpful to provide markers or bars to guide the reader to what conditions differ from each other.

We apologise for any lack of clarity. Where applicable, we have displayed key statistically significant results above or beside the relevant figure panels. However, due to our use of a 2x2x2 (genotype x exercise x age) linear mixed model design, we are not testing for statistical differences between individual columns or adjacent column pairs. Therefore, it is not feasible to add the labels as the reviewer suggested, as this would require a large area of complicated lines above each figure to indicate the relevant bars being compared for each main effect, and displaying the interactions would be yet more complex. Instead,

we have listed the significant main effects and interactions above the figure panels, providing the full statistical results in supplementary material.

Additionally, in Figures 5 and 6, vessel type is included as an extra factor in the statistical model. For these figures, statistical results could not be linked to individual panels as the model tested data presented across panels representing data from different vessel types. However in response to this point and similar points made by other reviewers, we have attempted to improve clarity by merging these figures, which were previously separate panels for each vessel type, into a single plot (5D,G; 6E; Supp. Fig. 4) and included the statistical results for each panel above the respective figures.

We would appreciate the reviewer's feedback on whether this modification facilitates the interpretation of our results, as now the plots themselves are more complicated, but it is easier to see at a glance what are the statistically significant results. If the reviewers feel this benefit is outweighed by the additional complexity of the plot, we can revert the figures to their original format.

9. Although the reader is referred to the supplemental tables for exact n-numbers, it would be informative to at least include an n-number range in the figure captions, particularly because some of the data is presented as n = number of mice, and n = other measure such as tissue slices, which does not inform about number of mice used. This needs to be more transparent in the figure captions.

We appreciate this valuable suggestion from the reviewer and have now included the ranges (e.g., number of mice, and measures such as cells or vessels, as applicable) in each figure legend. Initially, we attempted to break down these ranges per condition, but found that this approach made the information somewhat confusing and difficult to follow. Therefore, we opted to present the data as a range within the figure legends, while providing the detailed breakdown of full N values in the Supplementary document.

Minor:

10. Some figure captions seem to include a lot of exposition/interpretation about the results presented which would be more appropriate to include in the main text.

We take the reviewers point that there was too much explanation in some figure legends, particularly Figure 1, so have edited the content to reduce this, while retaining some information in the figure legends to allow them to be read somewhat independently from the main text.

11. Age groups are not labeled in the figure 3 bar graphs.

We apologise for the omission. We have added in the labels.

12. Fisher's Z score does not appear to be described in the methods.

We apologise for the omission and have now added the Fisher's Z transformation as Equation 1 (now line 941).

Reviewer #2 (Remarks to the Author):

This novel study by Anderle et al reports on understandings on the impact of the APOE4 genotype with the specific lifestyle contribution of exercise. The study builds on work on understanding the impact of APOE4 by Bonnar et al who reported impaired vascular responsiveness and vasomotion in awake mice. The experiments in this study were all conducted in awake mice, therefore providing an important contribution to the field and in vivo experimental community, free from confounding effects by anaesthesia. The major claim that a lack of exercise in association with the APOE4 genotype is detrimental to cerebral vascular reactivity and function is an important one, on which further research is needed to identify the specific mechanisms, as the authors have identified in the discussion.

The experimental design is impressive, and the range of data collected/techniques used leads to a thorough conclusion important and of interest to the cerebral vascular field.

We thank the reviewer for their positive assessment of our work.

One general comment is the age range (2/3 and 6 months) used to create age as a variable, as according to https://www.jax.org/research-and-faculty/research-labs/the-harrison-lab/gerontology/life-span-as-a-biomarker# (Figure V.3) 3-6 months still equates to 'mature adult' of 2-30 years, making it tricky to use age as a variable. Perhaps a solution could be for the authors could be more explicit about the age equivalent in human years, or refer to this variable in the text as duration of exercise?

This is a very good point. We didn't want to imply that this is an ageing study, but using age as a variable does create that sense. Instead, we agree that using "duration of exercise" as

the key concept for this variable is much better. Throughout the manuscript, we have now changed the phrasing to reflect not age but time since mice had differential wheel access and called this variable "duration" in all statistical reports.

Did the authors record the weight of the mice to check if exercise was causing the mice to be leaner/whether there were any differences in weight between the 2 genotypes?

We thank the reviewer for raising this important point. We did record the weight of some of the mice involved in the study. Our analysis shows that there is no difference in body weight due to genotype and exercise, but that there is a significant effect of age ($p = 3.03e-07$), with mice weighing significantly more at older age, as expected. As we think this information is important, we have now included the plot in Fig.1 and incorporated the results of the statistical test in the main document and the supplementary material.

Please see below for specific comments:

-Line 43: please could the authors add a reference for the statement about the weak binding of APOE4 to LRP-1 as this is not fully described in reference 6 (Montague et al) or use a more specific reference (eg Halliday et al 2016 PMID 25757756)

We thank the reviewer for noticing this mistake. Upon further inspection of the literature we could not find a specific paper that proved that APOE4 binds more weakly to LRP-1 receptor to cause the disinhibition of the CypA/MMP9 pathway. The paper the reviewer suggests does not interrogate LRP1 binding and shows only that LRP1 reduction coincides with an increase in CypA and MMP9 that is larger in APOE4 carriers. Therefore, we have now changed the sentence in the manuscript as: "APOE4 is known to affect the brain vasculature, disrupting blood brain barrier (BBB) function (e.g.[6]) likely via LRP-1 and the cyclophiliin A-matrix metalloproteinase 9 (CypA/MMP9) pathway."

-Line 71: please could the authors state how they decided on the duration of housing with an exercise wheel for '1-2 or 4-5 months'

Thanks to the reviewer for highlighting the need for an explanation regarding the duration of exercise. We have now included the following statement at line 798 that clarifies our reasoning behind this choice:

"Periods of exercise and in vivo imaging time points were chosen to match the age at which recordings were conducted in our previous study. "

-Figure 1a: please could the authors change the age in weeks at time of surgery to months to match the latter three timepoints for clarity

This has now been changed. We use the average 2 months value for consistency/ease of understanding. Some mice that did not have exercise recorded had slightly later surgery (<3 months) and these mice are listed in the cohort details in the methods.

-Please could the authors state why exercise and neurovascular function were recorded separately at 2 and 3 months, but together at 6 months?

We thank the reviewer for rightly highlighting the need for an explanation regarding the choice of voluntary exercise recording time point at 2-months of age. We have now included the following statement in our manuscript at line 799:

"Voluntary exercise was recorded at the start of the experiment at 2-months of age to capture the highest activity levels performed by each mouse. Mice are more active at 2 months than at older ages and physical exercise at 2 months have the greatest changes in transcription of genes involved in cerebrovascular remodelling.[25]"

-Figure 1 f and g: if possible, please could the authors consider putting in an axis break on the y axis to highlight the difference in distance run between 2 months and 6 months?

We have now inserted a figure of exercise distances plotted on the same scale, as Supp. Fig. 1 and referenced it in the text so that the reader can more easily see the differences in exercise level across the experiment.

-Figure 1 h: according to supplementary table 1, the n number for 2 months E3EX is n=3, the figure legend suggests that each dot represents an animal, but there are more dots on the graph – please could the authors clarify what the dots represent?

We apologise for the mistake in the figure description and thank the review for spotting it. Each dot represents the distance travelled each day of the recording by each mouse. As the recordings were carried out for 3-5 days, each mouse would have multiple daily exercise data points. The caption was amended in line 116 as follows:

"Dots are daily distances travelled by each mouse during each day of the recording session."

In addition, the number of animals and recordings for each group were added to the figure legend in response to a point raised by Reviewer 1, and we believe will also help in communicating what individual points represent.

-Line 150, figure 2 legend: please could the authors clarify if each brain slice is from one animal

We thank the reviewer for requesting a clarification regarding the number of slices per animal that were used to measure vascular density. As mentioned above, we have now included the number of slices and animals in the figure legend in lines 173-186, as requested by Reviewer 1, and we hope this will provide clarity regarding the number of slices and animals involved in this analysis. (N: E3EX 9 slices from 4 mice, E3NOEX 7 slices from 4 mice, E4EX 7 slices from 3 mice, E4NOEX 7 10 slices from 4 mice). So for each animal, 1-3 slices were used to measure vascular density.

-Lines 139 – 142: please could the authors correct the figures referred too (eg flux on line 139 should refer to Fig 2 c instead of Fig 2 d)

We apologise for the labelling error. These figure references have been corrected.

-Please could the authors clarify which region the capillary density was measured in, as in line 176, it states hippocampus and line 127 visual cortex. Please also check line 149 for the description of the region in the figure legend.

We apologise for the confusion. As mentioned above in response to the first reviewer, in an earlier draft we included capillary density data from the hippocampus, but as the focus of all the in vivo work is the visual cortex, and we couldn't therefore investigate the small genotype effect we saw in the hippocampus, we excluded those data from this current draft. We are sorry that we had failed to remove all the references to those data in the manuscript and have now reworded the lines the referee mentions to remove reference to the hippocampus data.

-Please could the authors match the x axis on Fig 3c for clarity?

Thank you for noticing this error. We have now corrected the missing axis labels.

-Line 234: please could the authors check the p value for the main effect of exercise on responsiveness as the values in the supp table state p=0.0236 rather than p=0.017, and also the p value for genotype line 235.

Many thanks to the reviewer for highlighting this mismatch, however, upon close inspection, we could not detect any mismatch between the p values presented in lines 234-235 (now lines 279-280) and what is reported in Supp Table 4 (now Supp Table 5). We are sorry we can't find the problem. Please can the reviewer point us more precisely to the issue and we will be very happy to correct it?

-Line 235-236: please could the authors clarify whether the effect by APOE4 impacted by exercise is across vessel type or just one vessel type (as the p value is not in the supplementary table)

We thank the reviewer for raising this point and we apologise if this was not clear. The statistical output reported in Supp. Table 4 (now Supp. Table 5) shows that there was no significant interaction between genotype, exercise and vessel type, which suggests that the interaction between APOE and exercise on the size of responses is similar across vessel types. We are not sure what p value we are missing but are happy to further correct if we are misunderstanding what the reviewer asks.

-Lines 247 – 248: please could the authors check the p values as they do not match up to the p values in the figure or supplementary table

We thank the reviewer for the comment, however when checking we do not find a mismatch between the p values reported in lines 247-248 (now lines 296-298), i.e. the p-value for RBCV time to peak during stim, and the p values reported in Supp Table S3 (now Supp Table S4). However, we have noticed that in Supp Fig 3k (now Supp Fig 4e) we didn't include the p value in the graph, which we have now amended. Again, if we have misunderstood what the reviewer had noticed then we are very happy to correct it.

-Lines 255-270: The figure legend for Figure 4 is a little unclear; please could the authors check the corresponding letters?

We apologise for the mistake. We have amended the text to increase clarity and to reference some figure panels that we had missed out.

-Figure 5: the results from the NVCi calculations are very interesting – would it be possible for the authors to add p values to the graphs to highlight the effect of exercise and genotype across the vessel types?

We are very glad that the reviewer finds these results interesting, as do we. Unfortunately, we cannot add p values to the different graphs as originally formatted, as they suggest. To

analyse these data, we used a single linear mixed model across all the data in the 4 panels referenced (5e-h). The relevant statistical results (interactions between vessel type and the different main effects) therefore apply across all these panels and cannot be linked to a single panel as this figure was originally plotted. However, in response to this point and other similar points made by other reviewers, we have now replotted these data showing all vessels on the same figure panel, so that the statistical results can be displayed on the same graph. This is now Figure 6e.

-Figure 6: please could the authors clarify whether the comparison of distance run at 2 months with data from mice at 6 months are between the same mice ie are these the same mice being compared throughout the study?

We thank the reviewer for raising this important point. We have indeed recorded the exercise from the same mice, whenever possible. We have now included the following sentence in line 709 to make the reader aware of this:

"Activity recordings were conducted on the same mice, whenever possible, at 2-months and 6-months of age, i.e. at the start and after 4 months of differential wheel access (N=1 mouse died before the second time point and N=2 mice had to skip the first recording time point because of COVID-19 restrictions)."

-When referring to supplementary figure 6 between lines 342 – 357, please could the authors add the letter for each part of the figure referred to for clarity?

We apologise for the oversight and have added letters to the Supplementary Figure references where these were missing.

-Discussion Line 367: the phrase 'different levels of voluntary exercise' could potentially be misleading as it could suggest easy/difficult exercises; could the authors consider reframing eg by saying 'different periods of exercise' or something similar?

We have reworded the sentence as shown below to avoid this confusion (now line 472).

"To understand how APOE4 increases the risk of developing AD and its effect on neurovascular function, including its interaction with lifestyle factors, we studied neurovascular function in APOE4-TR and APOE3-TR mice that underwent different durations of voluntary exercise."

-Discussion Line 443-444: the references are in a different format (not numbered). Also, for reference 42 at the end of line 444, as the results suggested that exercise was not beneficial for the capillary bed, please could the authors comment on the implications of that reference (where ROS stimulated ET-1 constricts capillary pericytes) in the context of their findings?

We apologise for the mistake and have now correctly inserted the misformatted references. We have also reordered and amended the paragraph referred to, as follows, in order to more clearly link the potential impact of ROS and ET-1 on capillary pericytes and how this could be modulated by exercise levels (line 560).

"Exercise can directly reduce plasma levels of the potent vasoconstrictor endothelin-1 (ET-1) by increasing pulsatile-induced shear stress via increased blood flow[41] and by reducing the synthesis of its precursor, big-ET[42]. In addition to altering ET-1 levels directly, exercise may act via altering levels of ROS. Exercise can reduce superoxide production by reducing the activity of NADPH oxidase and increasing the activity of the superoxide dismutases SOD1 and SOD3[43]. ET-1 production is promoted by ROS, which are found at higher levels in APOE4 mice [7,13] and ROS-stimulated ET-1 production constricts capillary pericytes[26], thus an exercise-mediated reduction in ROS could reduce ET-1 production and reduce capillary pericyte constriction, which will promote blood flow[44]. Indeed, reducing levels of superoxide can improve blood flow, as the superoxide scavenger MnTBAP ameliorated the large decrease in CBF observed in acute surgical preparations of APOE4 mice[7]."

-It is interesting that despite the decrease in exercise activity at 6 months compared to 2 months (Figure 1), there is still preservation of neurovascular coupling responses in the APOE4 mice (Figure 5). Do the authors have any suggestions as to why this may be the case?

The reviewer raises a really interesting point. To rephrase the point slightly (and please do correct us if this is different to what the reviewer meant), it is interesting that neurovascular coupling (in arterioles) is preserved in the exercising APOE4 mice at 6m, even though they are not exercising as much as they were at 2m and indeed are exercising less than the APOE3 mice.

This point by the reviewer led us to dig deeper into the relationship between neurovascular coupling and exercise, which revealed several interesting observations.

We split the analyses of correlations with the amount of exercise into two groups: correlations of exercise with measures of basal neurovascular function and with measures of neurovascular coupling. This revealed a stark difference between the relationship of basal and neurovascular coupling measures with exercise and how these changed over time. Only basal measures showed an increase in correlation from 2m to 6m of age (i.e. from the start of the experiment to 4 months of differential exercise exposure), such that mice that exercised more showed increased sO2, CMRO2, HbT, CBF and vasomotion at 6m but not at 2m, when these measures were in fact lower in mice that exercised more. Conversely, overall, neurovascular coupling measures were correlated with exercise level even from 2-3m (see Figure below, now Figure 7). Correlations were all performed on normalised data, so these analyses don't take into account the lower amount of exercise overall at 6m, only the relative amount of exercise across the cohort. Despite the decrease in the actual amount of exercise, therefore, the mice that exercise most show the strongest NVC at both 2m and 6m .

Furthermore, when we recalculated the correlations using only mice from each genotype separately and split these results into correlations of exercise with basal or NVC measures, it became apparent that the APOE4 mice showed a lower correlation between exercise and measures of basal neurovascular function than APOE3 mice, but there was no genotype difference in their correlations between exercise and neurovascular coupling measures. This latter result reflects the observation that neurovascular coupling in the APOE4 mice can be rescued by physical activity (except in capillaries). Importantly, however though we found no significant effect of genotype on individual measures of basal neurovascular function (Fig.2,3) this new analysis suggests that, in fact, basal neurovascular function is negatively impacted by APOE4 genotype, as it is less amenable to improvement by physical activity than in APOE3 mice.

Considering the reviewer's original question, these extra analyses suggest that NVC is as related to ongoing levels of exercise at 6m as it is at 2m for both APOE3 and APOE4 mice. As the actual amount of exercise has dropped by 6m, if exercise is causing this correlation, then less exercise must be required to sustain NVC at 6m than at 2m. Alternatively, the key causative feature may not be the degree of exercise per se, but another factor (e.g. engagement with the environment) that is reflected in their relative but not absolute degree of exercise. In this case, mice could run less over time without being less engaged with their environment, and therefore continue to benefit from the impact of relatively increased activity.

The wheel represents a form of environmental enrichment, so the amount of running could reflect the degree of engagement with this more interesting environment. We therefore recalculated the correlations using mice either from the exercise or no exercise groups. In the no wheel group, there was no longer an increase over time in the degree of correlation between neurovascular function and degree of exercise (see Figure below, now

Supplementary Figure 6). This suggests that the interest value of the wheel may be contributing to the beneficial effect of physical activity, supporting the idea that degree of engagement may be an underlying factor driving the relationship between exercise and improvised neurovascular function.

It should be noted, however, that reducing the number of animals used to generate the correlation coefficients by splitting them by genotype or exercise group, reduces the number of points used to calculate the correlation coefficients and therefore increases the uncertainty in these measures (see the increase in 95% CIs in the figures below). Therefore further experiments and more mice would be necessary to better evaluate correlations in the genotype and exercise subgroups.

A further thing to note is that capillary neurovascular coupling remained impaired in the APOE4 mice with access to a wheel. This could be because capillaries have a higher requirement for exercise, or that their function is independent of exercise. The low correlation coefficient of capillary NVCi with exercise argues for the latter interpretation (see Fig 6e). Instead, we think that the impact of exercise may act on regulatory pathways such as NO signalling that are more important for regulating arteriole than capillary function.

We have incorporated these new analyses into Figure 7 and Supplementary Figure 6 and added text to the results (lines 398-420) and discussion sections (lines 649-658).

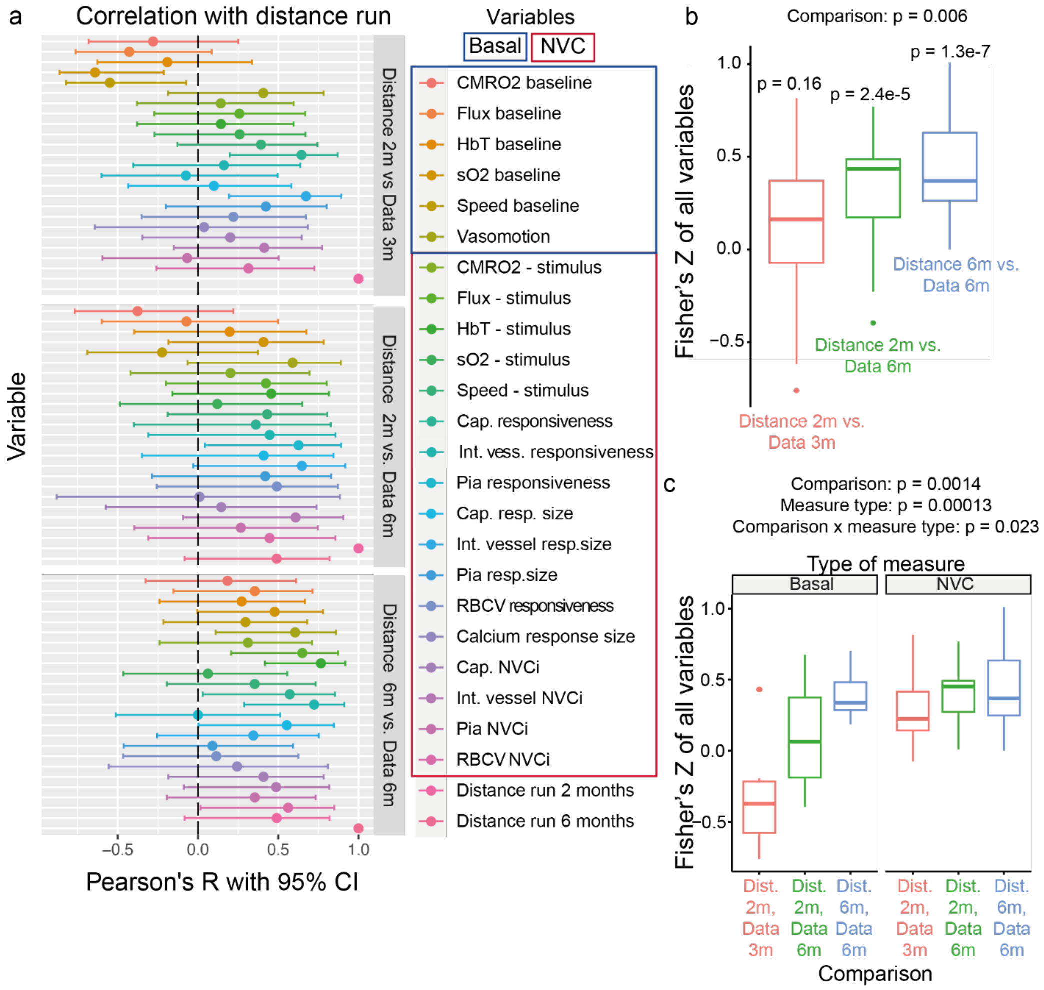
a
Correlation with distance run
Variable
Distance 2m vs Data 3m
Distance 2m vs. Data 6m
Distance 6m vs. Data 6m
-0.5
0.0
0.5
1.0
Pearson's R with 95% CI
Variables
Basal
NVC
CMRO2 baseline
Flux baseline
HbT baseline
sO2 baseline
Speed baseline
Vasomotion
CMRO2 - stimulus
Flux - stimulus
HbT - stimulus
sO2 - stimulus
Speed - stimulus
Cap. responsiveness
Int. vess. responsiveness
Pia responsiveness
Cap. resp. size
Int. vessel resp.size
Pia resp.size
RBCV responsiveness
Calcium response size
Cap. NVCi
Int. vessel NVCi
Pia NVCi
RBCV NVCi
Distance run 2 months
Distance run 6 months
b
Comparison: p = 0.006
p = 0.16
p = 2.4e-5
p = 1.3e-7
Fisher's Z of all variables
1.0
0.5
0.0
-0.5
Distance 2m vs. Data 3m
Distance 2m vs. Data 6m
Distance 6m vs. Data 6m
c
Comparison: p = 0.0014
Measure type: p = 0.00013
Comparison x measure type: p = 0.023
Type of measure
Basal
NVC
Fisher's Z of all variables
Dist. 2m, Data 3m
Dist. 2m, Data 6m
Dist. 6m, Data 6m
Comparison

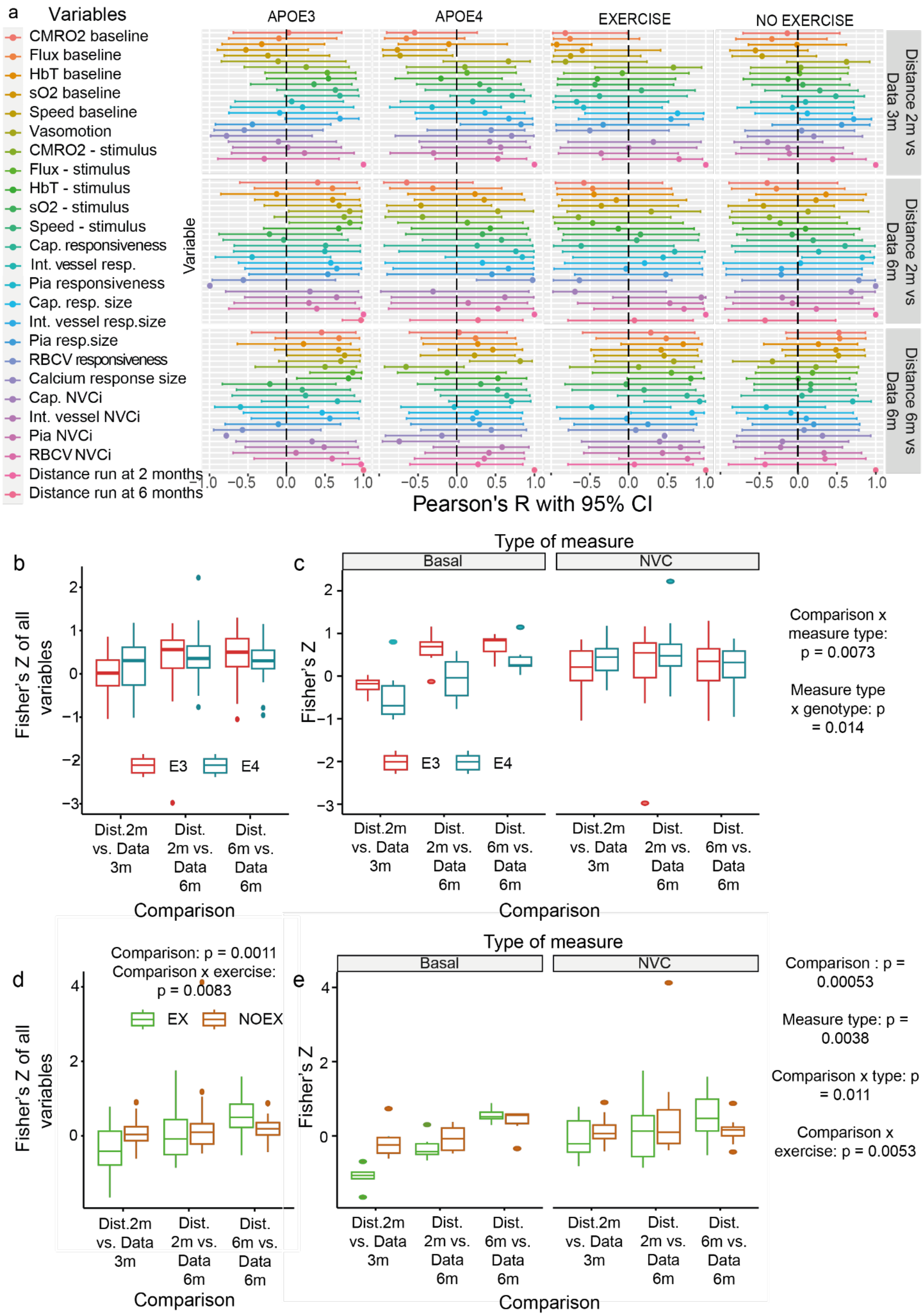
a
Variables
CMRO2 baseline
Flux baseline
HbT baseline
sO2 baseline
Speed baseline
Vasomotion
CMRO2 - stimulus
Flux - stimulus
HbT - stimulus
sO2 - stimulus
Speed - stimulus
Cap. responsiveness
Int. vessel resp.
Pia responsiveness
Cap. resp. size
Int. vessel resp.size
Pia resp.size
RBCV responsiveness
Calcium response size
Cap. NVCi
Int. vessel NVCi
Pia NVCi
RBCV NVCi
Distance run at 2 months
Distance run at 6 months
APOE3
APOE4
EXERCISE
NO EXERCISE
Variable
Distance 2m vs Data 3m
Distance 2m vs Data 6m
Distance 6m vs Data 6m
Pearson's R with 95% CI
b
Fisher's Z of all variables
E3
E4
Dist.2m vs. Data 3m
Dist. 2m vs. Data 6m
Dist. 6m vs. Data 6m
Comparison
c
Type of measure
Basal
NVC
Fisher's Z
Comparison x measure type: p = 0.0073
Measure type x genotype: p = 0.014
d
Comparison: p = 0.0011
Comparison x exercise: p = 0.0083
EX
NOEX
e
Comparison : p = 0.00053
Measure type: p = 0.0038
Comparison x type: p = 0.011
Comparison x exercise: p = 0.0053

-Line 738: the figshare link for processed data is broken.

We have checked the figshare link which appears to be working now.

Reviewer #3 (Remarks to the Author):

This manuscript examines the neurovascular response in APOE 3 and 4 mice with or without exercise. Numerous variables were measured yet clarity on the mechanisms related to APOE4 and vascular responses were not overtly elucidated. The current manuscript adds to the available literature and provides some modest advances. While the results are perhaps not as straightforward, the authors should be commended for the significant data collection and analyses. The supplementary details are also appreciated and extensive.

We thank the reviewer for their positive comments. We apologise that a detailed mechanistic breakdown is beyond the scope of this study.

1) While sedentary and APOE4 status resulting in worse outcomes is not too surprising, the current manuscript attempts to synthesize their findings cogently, but given the numerous variables assessed, it is difficult to digest the findings in total. The authors should consider a summary Table of sorts that summarizes the many findings to simplify for future readers the key aspects of the reported study.

We kindly thank the reviewer for this suggestion. We have now made a summary figure with the main results presented in our manuscript, which we have incorporated in a timeline of events that can happen after vascular dysfunction takes place. We hope this will add clarity and aid the interpretation of our results. The figure was inserted in the manuscript as Fig. 8 and as presented here:

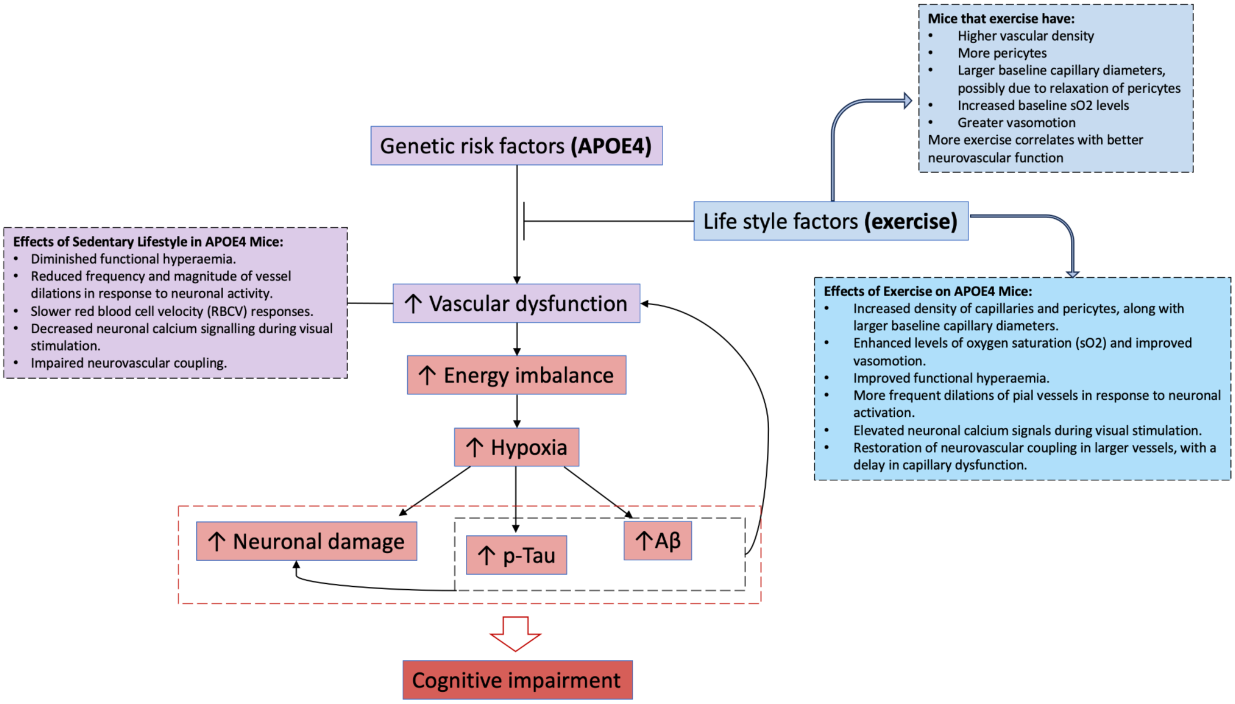

Figure 8: Summary of results - Genetic risk factors (APOE4) and lifestyle factors (degree of physical activity) act synergistically to cause vascular dysfunction. Our results indicate that genetic risk factors, such as the APOE4 genotype, can cause early vascular dysfunction, which is worsened by a sedentary lifestyle (purple panels). Instead, having an active lifestyle can improve measures of vascular function, even when APOE4 genetic risk factors are present (blue panels). Early vascular dysfunction can lead to an increase in energy imbalance, due to poor vascular responses to increased neuronal activity. As a consequence, less nutrients and oxygen are delivered to the brain. Speculatively, this could eventually lead to hypoxia, producing neuronal damage and promoting the accumulation of amyloid-b and p-Tau. These changes in turn can further increase vascular dysfunction and damage neurons (pink panels), setting up a detrimental cycle that culminates with the onset of cognitive symptoms (red panels). Early interventions, such as exercise, may therefore be critical to delay or prevent vascular dysfunction and halt the cascade of events that lead to dementia.

2) As the postmortem data vessel density was acquired from a previously published cohort, this should be directly noted in the results. In the present format this information is only available at the very end of the manuscript. Along similar lines in Fig 2 legend, there is reference to V1 and hippocampal data but it is not clear what region is being shown in Fig 2b.

We thank the reviewer for raising the point regarding the vascular density data being previously published. We did clearly state the data from the different cohorts, and what

contributed to a previous publication, in the Methods as well as the Discussion, but we agree this should also be stated in the Results section. We have now added the following sentence in line 788, which we hope will provide more clarity:

"The data from mice with access to an exercise wheel have been previously published[10]. "

Regarding the second point made by the reviewer, we apologise for the mistake, As mentioned above in response to the first two reviewers, this sentence was erroneously kept from a previous version of the manuscript that included capillary density data from the hippocampus as well. We decided to remove this data from the final version of the manuscript as the in vivo data is conducted in the visual cortex and we couldn't investigate the small genotype effect that we found in the hippocampal data. We are deeply sorry for not removing all the references to the hippocampus capillary density and we have now reworded the figure caption to match what is presented in the figure.

3) Fig 3 – Reading the results, the statistical results are noted in the text, but unlike Fig 2 with extensive statistical notation in the figure, figure 3 is mostly devoid of these notations. It is noticed also in other figures. Is there a reason for not being consistent across figures? It would be great to include all statistical notations in the figures. It is appreciated that all the details are in the supplement but would be beneficial to include across all figures.

We appreciate the reviewer's observation. As mentioned in our response to a similar comment from Reviewer #1, the challenge arises because the statistical model in some figures (previously Fig. 4 and Fig. 5) incorporates data from multiple panels representing different vessel types. As a result, the findings from this analysis cannot be attributed to a single panel.

To improve clarity, we have now combined the previously separated panels by vessel type into a unified plot (now Fig 5D,G; 6E; Supp. Fig. 4) and displayed the relevant statistical results above each panel. While this allows us to present the significant results more clearly, it may slightly obscure the overall pattern of the findings.

We value the reviewer's feedback on whether this adjustment enhances the interpretation of the results. If not, we are open to reverting to the original figure layout.

4) In several figures there are image or histological examples of the anatomy (ie Fig 4 b, f, j,n).. For completeness it would be nice to know what group these images came from - ie APOE3 Ex for example. See Fig 6a as well.

Many thanks to the reviewer for raising this point. We have now added in the figure captions the information regarding which group these histological images came from.

5) The extensive correlations reported and shown in Fig 6 clearly indicate a positive trend in many of the features assessed. The supplement also breaks this down by genotype. But what appears to be missing is a breakdown by genotype X exercise groups. Not sure if this was an oversight or if this was purposely not included. I would recommend adding this to the supplement as a minimum.

This was purposely not included because, to calculate the correlations for subgroups of mice, the correlations have to be recalculated using only the number of mice in that group. However not all mice used in the group mean analyses had exercise recorded. This means that when splitting by genotype and exercise group, the N used to calculate each correlation is very small, meaning there is a lot of uncertainty in the value of the correlation coefficients, and often there were too few (<=3) mice to allow 95%CI to be calculated. We have conducted the analyses, including the new split by NVC and basal flow measures we performed in response to a previous point, as shown in the figure below. There is a significant interaction between the comparisons across genotypes and exercise, but this is clearly driven by a very low correlation between the distance run at 2 months and the data collected at 6 months in the APOE4 NOEX group. However, inspecting the plot of the correlation coefficients across different measures demonstrates that many values are missing in this group and where there are values, the confidence intervals span the entire possible range. Therefore, we are not at all confident that the coefficients are meaningful, and would prefer to omit this analysis.

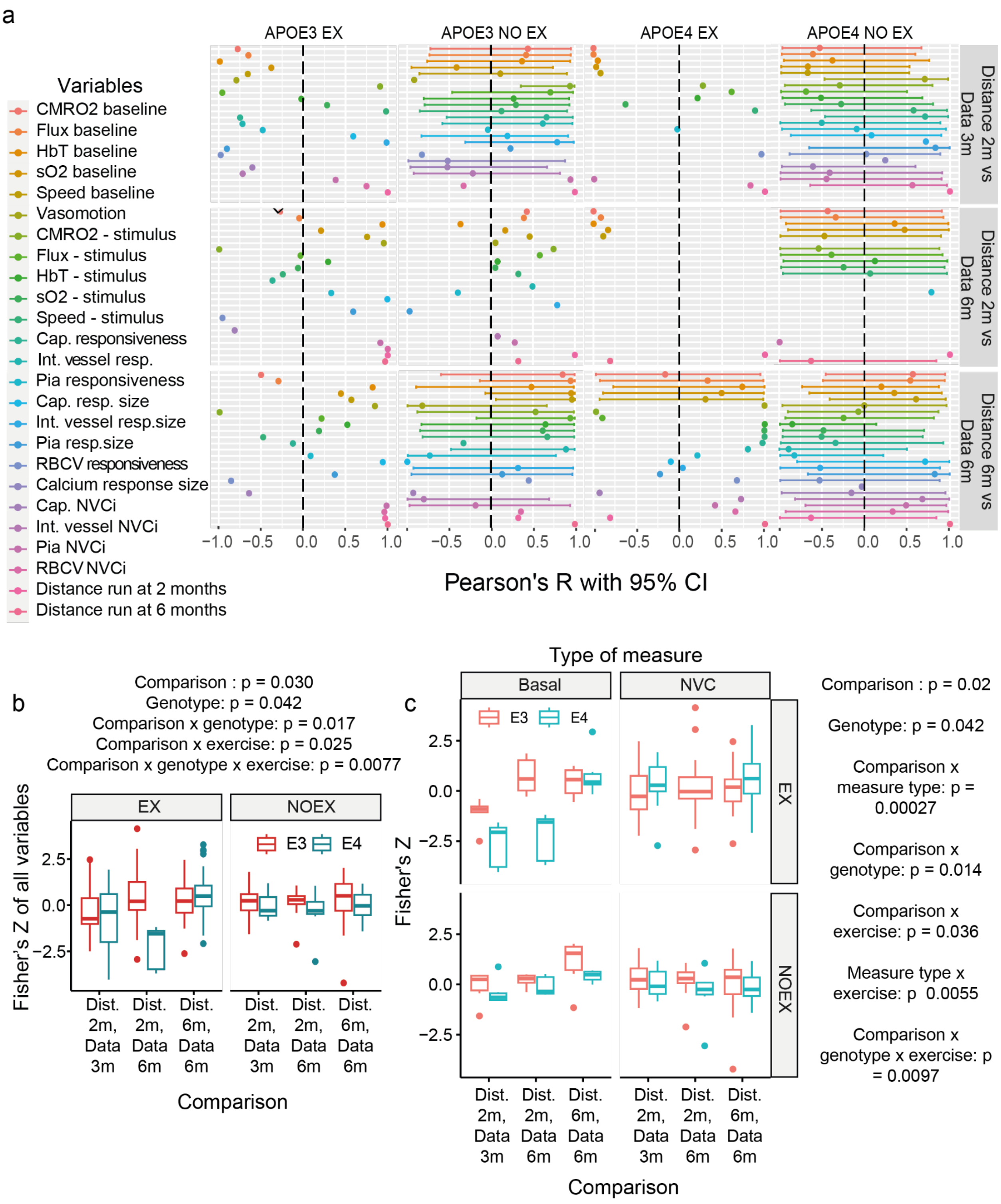
a
APOE3 EX
APOE3 NO EX
APOE4 EX
APOE4 NO EX
Variables
CMRO2 baseline
Flux baseline
HbT baseline
sO2 baseline
Speed baseline
Vasomotion
CMRO2 - stimulus
Flux - stimulus
HbT - stimulus
sO2 - stimulus
Speed - stimulus
Cap. responsiveness
Int. vessel resp.
Pia responsiveness
Cap. resp. size
Int. vessel resp.size
Pia resp.size
RBCV responsiveness
Calcium response size
Cap. NVCi
Int. vessel NVCi
Pia NVCi
RBCV NVCi
Distance run at 2 months
Distance run at 6 months
Distance 2m vs Data 3m
Distance 2m vs Data 6m
Distance 6m vs Data 6m
Pearson's R with 95% CI
b
Comparison : p = 0.030
Genotype: p = 0.042
Comparison x genotype: p = 0.017
Comparison x exercise: p = 0.025
Comparison x genotype x exercise: p = 0.0077
EX
NOEX
E3
E4
Fisher's Z of all variables
Dist. 2m, Data 3m
Dist. 2m, Data 6m
Dist. 6m, Data 6m
Comparison
c
Type of measure
Basal
NVC
E3
E4
EX
NOEX
Fisher's Z
Dist. 2m, Data 3m
Dist. 2m, Data 6m
Dist. 6m, Data 6m
Comparison
Comparison : p = 0.02
Genotype: p = 0.042
Comparison x measure type: p = 0.00027
Comparison x genotype: p = 0.014
Comparison x exercise: p = 0.036
Measure type x exercise: p 0.0055
Comparison x genotype x exercise: p = 0.0097

6) The discussion spends considerable time on several putative mechanisms that might underlie the findings in the manuscript, including NO (line 458). What is missing is that the source of the NO (vascular, vs neuronal etc) is likely a critical component to this discussion. Could the authors revise to clarify these interactions in light of their results.

We have amended the paragraph on NO to include the likely sources of NO (both eNOS and nNOS are likely modulated by exercise and genotype), as copied below:

"Adding a wheel to the cage manipulates the physical activity of the mice, but also adds environmental enrichment. Environmental enrichment is known to improve brain and cognitive function models of ageing and disease and, like exercise, can increase NO levels[60–62]. Dissecting the relative contributions of increased physical activity and cognitive stimulation of environmental enrichment is challenging and requires tracking home cage activity as we have done here. Because activity level was correlated with both basal and neurovascular coupling measures, physical activity is likely a major driver of the alterations in neurovascular function that we observe. However, in our experiments, the "no wheel" group showed a smaller increase over time in the correlation between physical activity and neurovascular function than the "wheel" group. This may mean that cognitive stimulation provided by the wheel modifies the relationship between physical activity and neurovascular function. Future experiments with larger group sizes to better capture correlations in subgroups are needed to test this hypothesis. "

7) Also there is no discussion as to the vascular density changes noted in other studies including VEGF mediated pathways and the relative lack of vessel density noted in the current models utilized herein. Also if there are vascular density changes in human APOE4 but not in the models utilized in this study, how do the authors reconcile relevance to human disease.

We apologise for this oversight. We have now added into the discussion the following statements, that should address the point raised by the reviewer:

"In addition to changes in neurovascular function, we also observed that exercise, but not genotype, increased capillary density and diameter near pericytes, with a potential increase in pericyte density. This corroborates previous work showing that exercise increases capillary density through angiogenesis[57]. This effect may be mediated by exercise-induced increases in lactate activating HCAR1 receptors located on pial fibroblast-like cells and capillary pericytes. Activation of HCAR1 leads to elevated VEGFA protein levels, ultimately promoting capillary angiogenesis[19].

APOE4 mice have also been shown to exhibit reduced VEGF levels[58], which could contribute to the reduced capillary density observed in some studies[7,8,59]. However, we did not observe a reduction in capillary density in APOE4 mice. The reason for this discrepancy is unclear. The previous studies included mice that were significantly older than the mice in our study[59], but also those of an equivalent age[7]. Methodological differences may contribute: For example, we quantified vascular density by filling the vasculature with a fluorescent gelatin, while other studies immunolabelled endothelial markers, so perhaps APOE4 reduces marker expression without affecting vessel perfusion. Whatever the reason, our data suggest that it is not necessarily the case that APOE4 expression leads to a reduction in vascular density. Indeed, no such reduction has, to our knowledge, been observed in healthy humans. Instead, APOE4-associated reductions in vascular density have only been documented in APOE4-positive Alzheimer's disease patients (e.g. [9]), where the effects could be due to an interaction of APOE4 with beta amyloid, tau or inflammatory pathology.

Together these reports suggest that APOE4 genotype can sometimes promote a reduction in vascular density, but this effect may become more apparent in the context of additional pathology. When this is the case, exercise-induced VEGFA production may help counteract these genotype effects, supporting healthy capillary density, but this would need to be specifically tested in new experiments."

Minor items:

1) In several locations within the manuscript, some references did not receive the numerical notification – ie line 169

We apologise for the mistake. We have now amended all of the references to be in the numerical format.

2) Also in the references, there are some references missing page numbers, others have doi's etc. These should be checked carefully.

We again apologise for the mistake. We have now amended all of the references to be in line with the Nature/Communications Biology referencing style, including journal issue and page numbers for all published references.

3) Some aspects of the study are greatly underpowered to study sex effects… and statistical testing is not warranted…. This should be made explicitly clear in the results as well as in the methods.

We thank the reviewer for raising this important point. We have now added the following statement at line 777 that provides an explanation as to why the analysis of sex effects was conducted and added into the supplementary materials even though the tests were heavily underpowered. We hope our data being available will allow these data to be included in any future metanalyses to interrogate sex effects with higher power.

"It is of note that the number of mice in some of the experimental groups was low when stratifying by sex, thus the analysis of sex effects was very underpowered. We nonetheless conducted these analyses in case any clear patterns of sex difference emerged that would warrant further follow up and in case future studies might benefit from this information."
